# The UNC5C T835M mutation associated with Alzheimer’s disease leads to neurodegeneration involving oxidative stress and hippocampal atrophy in aged mice

**DOI:** 10.1186/s13024-025-00850-z

**Published:** 2025-06-04

**Authors:** Devi Krishna Priya Karunakaran, Makenna Ley, Joanna Guo, Ammaarah Khatri, Katherine Sadleir, Jelena Popovic, Arun Kumar Upadhyay, Jeffrey Savas, Daniele Procissi, Jasvinder Atwal, Robert Vassar

**Affiliations:** 1 Department of Neurology, Feinberg School of Medicine, Northwestern University, Chicago, IL 60611 USA; 2 Mesulam Center for Alzheimer’s Disease and Cognitive Neurology, Feinberg School of Medicine, Northwestern University, Chicago, IL 60611 USA; 3 Center for Preclinical Imaging Core Facility, Feinberg School of Medicine, Northwestern University, Chicago, IL 60611 USA; 4 Department of Neuroscience, South San Francisco, Genentech, CA USA

## Abstract

Alzheimer’s disease (AD) is characterized by amyloid plaques, neurofibrillary tangles, and synaptic and neuronal loss. Recently, a rare autosomal dominant coding mutation, T835M, in the Un-coordinated 5c (UNC5C) netrin receptor gene was segregated with late-onset AD (LOAD). Overexpression of T835M in primary hippocampal neurons increased cell death in response to neurotoxic stimuli including beta-amyloid (Aβ) suggesting a mechanism by which T835M may confer increased risk of LOAD. However, the molecular mechanism of T835M-mediated cell death remained under explored. Toward this end, we generated a mouse T835M knock-in (*Unc5c*^*KI/KI*^) model and employed biochemical and histological analyses to understand the molecular mechanism of T835M-mediated pathogenesis in late onset Alzheimer's disease. We show that homozygous KI mice have significantly reduced hippocampal volume, increased ventricular volume, dendritic disorganization (CA1 region) and reduced UNC5C protein level by 12–18 months of age. Further, we show that the neuronal cell death is observed in the *Unc5c*^*KI/KI*^ mice by 12 months of age by TUNEL analysis and activated Caspase 3/7 assay. Proteomic analysis of hippocampal samples showed upregulation of oxidative stress and downregulation of chaperone proteins at 18 months corroborating the biochemical and histological results showing increased c-Jun N-terminal Kinase (JNK) phosphorylation, NADPH oxidase, and decreased Netrin1 levels. Moreover, *Unc5c*^*KI/KI*^ mice also show morphological changes in the astrocytes with increased number of branched processes, reduced GFAP levels, and significantly increased activation of microglia. Overall, these results suggest that T835M mutation causes neurodegeneration by creating an oxidative stress environment leading to synaptic degeneration and weakened astrocytes, thereby leading to neuronal cell death via apoptosis. Furthermore, to assess the effects of amyloid pathology on the mutation, we crossed *Unc5c*^*KI/KI*^ mice with *App*^*NL−G−F/NL−G−F*^ mice and observed an exacerbation of mutation-associated changes along with increased levels of Aβ_42_, suggesting that the T835M mutation increases the susceptibility of neurons to cell death and elevated Aβ_42_ levels, thus promoting AD pathogenesis. Understanding the molecular mechanism of cell death in regions susceptible to neurodegeneration such as the hippocampus could shed light on the players and pathways involved in cell death in AD pathogenesis and therefore could inform therapeutic approaches for AD.

**Supplementary Information:**

The online version contains supplementary material available at 10.1186/s13024-025-00850-z.

## Introduction

Alzheimer’s disease (AD) is a progressive neurodegenerative disease that is the 6^th^ leading cause of mortality in the US and is the most common cause of dementia in the geriatric population, accounting for 60–80% of all cases of dementia. It primarily affects the brain and causes gradual decline of cognitive abilities. Currently, about 6.7 million Americans are reported to live with AD and it is estimated to reach 13 million by 2050 (https://alz.org). The main hallmarks of the disease are amyloid plaques and neurofibrillary tangles, ultimately leading to progressive neuron loss. While drugs targeting AD pathologies, β-amyloid (Aβ) plaques and tau neurofibrillary tangles, are in development and anti-Aβ antibodies have been approved, therapies targeting other mechanisms of AD are desperately needed. Anti-Aβ antibodies slow but do not halt AD, benefit only early AD, and can have serious side effects. Therefore, discovering new safe drugs that benefit all stages of AD is of paramount importance. Understanding the molecular mechanism of cell death in regions susceptible to neurodegeneration such as the hippocampus could shed light on pathways involved in cell death, allowing development of AD therapeutics directed at novel targets.

Genome wide association studies (GWAS), Whole genome sequencing (WGS) and linkage studies have enabled the identification of genes that affect AD risk. About 95% of cases of AD are late-onset and sporadic and are associated with mutations in many genes. *Unc5c* (Uncoordinated C.*elegans* receptor 5c) is a candidate gene containing single nucleotide polymorphisms (SNPs) associated with late-onset AD [1]. The T835M mutation (rs137875858) in the hinge region of UNC5C was shown to increase the susceptibility of hippocampal neurons to cell death and segregated with late-onset AD (LOAD) [1]. Further studies by Hashimoto *et al*, showed that UNC5C T835M activated the cell death pathway in cell culture, suggesting a molecular mechanism involving JNK/PKD/NADPH oxidase signaling [2]. UNC5C acts as a chemorepellent for Netrin1, while its antagonist, deleted in colorectal cancer (Dcc), acts as a chemoattractant during axon guidance [3–5]. Besides its crucial role in various carcinomas, specifically in Colorectal cancer [6], UNC5C has also been implicated in various neurological disorders such as Autism spectrum disorder [7], Schizophrenia [8], and Parkinson’s disease [9]. Multiple SNPs in UNC5C have been shown to be associated with AD [1, 10–13]. UNC5C protein contains two Immunoglobulin domains (Ig), two thrombospondin domains (TS), a transmembrane domain (TM), a zona occludens-5 domain (ZU-5), a UPA domain, and a death domain (DD) [12]. It belongs to a class of transmembrane receptors called dependence receptors, which depending on the presence or absence of the ligand, could promote cell survival or induce cell death, respectively [14, 15]. Therefore, it is imperative to understand the molecular underpinnings of the T835M mutation in UNC5C-related brain atrophy and neuronal cell death to inform the potential of UNC5C as a therapeutic target for AD.

To gain deeper insights into the role of UNC5C in AD, here we analyze the CNS phenotype of UNC5C T835M targeted replacement mice (*Unc5c*^*KI/KI*^). *Unc5c*^*KI/KI*^ mice have age-related neurodegeneration, including reduced hippocampal volume, increased ventricular volume and reduced white matter connectivity beginning at 12–18 months of age. Moreover, the UNC5C T835M mutation results in decreased pre-synaptic but increased post-synaptic protein levels, dendritic disorganization in the hippocampal CA1 region, and increased apoptotic neuronal death. In addition, astrocytes and microglia in CA1 have reduced Glial fibrillary acidic protein (GFAP) levels and increased activation, respectively. Proteomic studies reveal increased oxidative stress proteins in the hippocampus and decreased chaperone proteins, along with increased c-Jun N-terminal Kinase (JNK) phosphorylation, NADPH oxidase, and decreased Netrin1 levels. To understand the effects of the UNC5C T835M mutation on AD pathogenesis, we generated mice that were homozygous for both UNC5C T835M and the APP targeted replacement, App^NL−G−F/NL−G−F^ (NLGF) [16], which develop amyloid plaques and synaptic loss by 6 months but no significant neuron loss. In NLGF;*Unc5c*^*KI/KI*^ double knock-in (dKI) mice compared to NLGF mice alone, we observed an exacerbation of UNC5C T835M-associated phenotypes, such as increased cell death, reduced hippocampal area, and decreased Netrin1 and GFAP levels at 6 and 12 months of age. Overall, these results suggest that the UNC5C T835M mutation causes neurodegeneration by increasing oxidative stress leading to synaptic degeneration and neuronal apoptosis, which are all worsened in the presence of cytotoxic stressors such as amyloid, therefore increasing AD susceptibility.

## Materials and methods

### Mice

Unless indicated, no significance was noted between the genders, and the data presented were the means of both male and female animals. UNC5C T835M KI (KI) mice were generated by Genentech Inc. (JA and RW) and acquired by Northwestern University. Briefly, the construct for targeting the *Unc5c* locus in mouse ES cells to generate the T835M KI allele, was made using a combination of recombineering, gene synthesis and standard molecular cloning techniques. The resulting targeting vector enabled insertion of the T835M point mutation in *Unc5c* exon 15 with an FRT-*Pgk1*-em7-Neo-FRT cassette inserted in intron 15 at genomic position mm10 chr3:141,827,785. The vector was confirmed by DNA sequencing, linearized with NotI and used to target C57BL/6 C2 ES cells using standard methods (G418 positive and gancyclovir negative selection). Positive clones were identified using PCR and TaqMan analysis and confirmed by sequencing of the modified locus. Correctly targeted ES cells were infected with Adeno-Flpo [17] to remove the selection marker with a single FRT site remaining, resulting in the final *Unc5c*^T835M^ KI allele (*Unc5c*^*KI/KI*^). Validated ES cells were injected into blastocysts using standard techniques, and germline transmission was obtained after crossing resulting chimaeras with C57BL/6 N females.

All animal work was performed in Northwestern University in accordance with Northwestern University Institutional Animal Care and Use Committee approval. The number of biological replicates for each experiment is specified in the figure legends. *App*^*tm3.1 Tcs*^*/App*^*tm3.1 Tcs*^ mice (NLGF mice (*App*^*NL−G−F/NL−G−F*^)) were obtained from Dr. Takaomi Saido, RIKEN Brain Science Institute, Japan, and NLGF homozygous mice were bred with *Unc5c* KI (*Unc5c*^*KI/KI*^) homozygous mice to obtain the double KI (dKI) mice [16]. *Unc5c* KO (*Unc5c*^*KO/KO*^*/Unc5c*^*−/−*^) mice were obtained as heterozygotes from Dr. Susan Ackerman at University of California, San Diego, and bred in-house to obtain the homozygotes [18]. Genotyping for KI allele was performed by Transnetyx using custom probes for Unc5c (WT and mutant specific probes) and standard probes for NLGF mice.

### Antibodies

The antibodies used were as follows: rabbit anti-β-actin (#926–42,210, LI-COR), mouse anti-BACE1 (3D5) (made in Vassar lab) [19], rabbit anti-BACE1 (#ab108394, Abcam), rat anti-MBP (#ab7349, Abcam), mouse anti-PSD95 (#K28/43, DSHB), rabbit anti-C1q (#ab227072, Abcam), rat anti-CD68 (#14–0681-82, Invitrogen), chicken anti-MAP2 (#ab5392, Abcam), rabbit anti-Cdk5 (#14145S, Cell Signaling), chicken anti-NeuN (#ABN91, Millipore), mouse anti–β-tubulin (Tuj1) (a gift from L. Binder), anti-synaptophysin (WB: mouse #ab8049, Abcam; IF: goat #AF5555, R&D Systems), rabbit anti-NADPH oxidase (#17,772–1-AP, Proteintech), rabbit anti-total JNK/SAPK (#9252, Cell Signaling), rabbit anti-phospho-JNK (#9251S, Cell Signaling), rabbit anti-PKD (#PA5-13,749, Invitrogen), mouse anti-SMI312 (#837,904, BioLegend), anti-Iba1 (WB: rabbit #ab178846, Abcam; IF: goat #NB100-1028, Novus), anti-GFAP (WB: rabbit #G9269, Sigma-Aldrich; IF: chicken #ab4674, Abcam), rabbit anti-GAPDH (#2118, Cell Signaling), rabbit anti-UNC5C (polyclonal antibody which was custom made by Proteintech Inc., against the C-terminal 400 amino acids of Unc5c protein), rabbit anti-Ab_42_ (#700,254, Invitrogen), rat anti-Lamp1 (#1D4B, DSHB), 3D6 mouse anti-Aβ monoclonal antibody [20] (gift of Dr. Lisa Conlogue, Elan Pharmaceuticals) (antigen is 1–5 N-terminal amino acids of Aβ_42_), Rabbit anti-Netrin-1 (#MBS821997, My Biosource Inc,.) and Human Netrin-1 Protein, His Tag (NEI-H52H3, Acro Biosystems). 1g of the purified protein was loaded to confirm the Netrin1 band around 85kD.

### Tissue extraction and immunoblot analysis

Mice were deeply anesthetized by intraperitoneal injection of xylazine (15 mg/kg) and ketamine (100 mg/kg), perfused with ice-cold phosphate-buffered saline (PBS) with phenylmethylsulfonyl fluoride (20 µg/ml), leupeptin (0.5 µg/ml), sodium orthovanadate (20 µM), and dithiothreitol (0.1 mM), followed by decapitation and brain removal. The hemibrain was dissected on ice into the cortex, hippocampus, and cerebellum, and then snap-frozen in liquid nitrogen and stored at − 80 °C. Tissues were homogenized in radioimmunoprecipitation assay buffer ((RIPA; 50 mM tris, 0.15 M NaCl, 1% octylphenoxypolyethoxyethanol (IGEPAL), 1 mM EDTA, 1 mM EGTA, 0.1% SDS, 0.5% sodium deoxylate (pH 8)), followed by sonication and centrifugation. All buffers contained protease inhibitor cocktail III (#535,140, Millipore) and Halt phosphatase inhibitor (#78,420, Thermo Fisher Scientific). Protein concentration was determined using bicinchoninic acid assay (BCA) assay (#23,225, Thermo Fisher Scientific). Equal amount of protein was separated under reduced and denatured conditions, transferred onto a polyvinylidene difluoride or nitrocellulose membrane, and developed using Pierce ECL (enhanced chemiluminesence) (Thermo Fisher Scientific) on a ProteinSimple FCR imager and Biorad imager. Chemiluminescent signals were quantified using AlphaView software (ProteinSimple) and Imagelab (Biorad).

### Immunofluorescence

Hemibrains were fixed in 10% formalin and preserved in 30% sucrose/PBS solution. Brains were sectioned as coronal sections at 30µm on freezing-sliding microtome and stained using the free-floating method. Sections were serially placed in a 12-well plate in a cryoprotective solution (1xPBS, 30% sucrose, and 30% ethylene glycol) and stored at − 20 °C until use. Immunofluorescence staining was performed by first washing sections three times in 1xTBS and then incubating sections in 16 mM glycine in 1xTBS for 1 h at room temperature. After 3 additional washes in 1xTBS, sections were blocked in 5% donkey serum in 0.25% Triton X-100 in 1xTBS for 2 h at room temperature. The sections were then incubated overnight in primary antibodies in a solution of 0.25% Triton X-100, 1% bovine serum albumin and 1xTBS at 4 °C. Alexa Fluor secondary antibodies (Invitrogen) were used at a concentration of 1:750. Sections were mounted using ProLong Gold (#P36934, Thermo Fisher Scientific) and imaged on a Nikon A1R or AXR laser scanning confocal microscope or Nikon Ti2 widefield microscope (Northwestern University Center for Advanced Microscopy).

### Terminal d-UTP nick-end labeling (TUNEL) Cell death detection

For cell death detection, sections were permeabilized with Triton X-100 for an hour, followed by a 1-h incubation of reaction mix from the In Situ Cell Death Detection Kit, TMR Red (#12,156,792,910 Roche, Sigma-Aldrich) at 37 °C following the directions on the user manual.

### Activated Caspase-3/7 Fluorescence assay

We assayed the activity of caspase-3/7 using Caspase-3/7 Fluorescence assay kit (Cayman chemical, cat # 10,009,135). Briefly, 90 µl of diluted hippocampal homogenates were added to a black 96-well plate. 10 µl of assay buffer was added to each well to assay for the endogenous activity of cleaved caspase-3/7. 100 µl of positive control of active Caspase-3 was added to a couple of wells as a positive control. 100 µl of the caspase-3/7 substrate solution was added to each well and incubated for 60–90 min. The relative fluorescence intensity was read at 535 nm.

### ELISA & MSD-ELISA

For NLGF and dKI hippocampal samples, we treated the extracts with Guanidine hydrochloride (7.2 μl of 2 mg/ml brain homogenates were added to 12.8 μl of freshly made 8.2 M guanidine hydrochloride (GuHCl); 82 mM Tris HCl (pH 8.0) (5 M GuHCl final) and mixed for three days on a nutator) and the samples were then diluted with the assay buffer and ELISA for Aβ_42_ (Invitrogen, cat# KHB3441) was performed according to instructions in user’s manual.

MSD-ELISA was performed on the hippocampal samples from NLGF and dKI mice at 6- and 12- months using V-PLEX Aβ Peptide Panel 1 (6E10) assay Kit (Meso Scale Discovery, cat# K15200E) following the directions on the user’s manual. Briefly, the hippocampal samples were treated with Guanidine hydrochloride as described above, to obtain the insoluble Aβ species. Samples were further diluted 128-fold with diluent-35 (from the kit) and assay was performed.

### MRI brain region volumetric analysis

Acquisition was done on a 7 Tesla Bruker Clinscan MRI using a 3D multiple echo GRE sequence with isotropic spatial resolution. After realignment, to avoid errors in volume/morphological estimation associated with different head position, data was extracted and comparison between relevant brain regions (ventricle and hippocampus) across cohorts was done by averaging the regional volumes extracted via segmentation and normalized using each whole brain volume. The original 3D high resolution MR images (110 μm) were used to derive these quantities. Each segmentation was implemented using a semi-automated approach which combined the use of the threshold automatic segmentation tool contained in IT-SNAP followed by manual corrections using a graphical pen and a dedicated Wacom tablet to enable accurate delineation of anatomical regions. The manual corrections were implemented to avoid artifacts from automated segmentation and to reliably capture volumetric data from the same exact regions across different subjects. Delineation was performed by two experienced neuroimaging scientists (DP) using a standard mouse brain atlas as reference guide for identification of relevant anatomical regions.

### MRI cortical thickness analysis

**Fig. 1:**
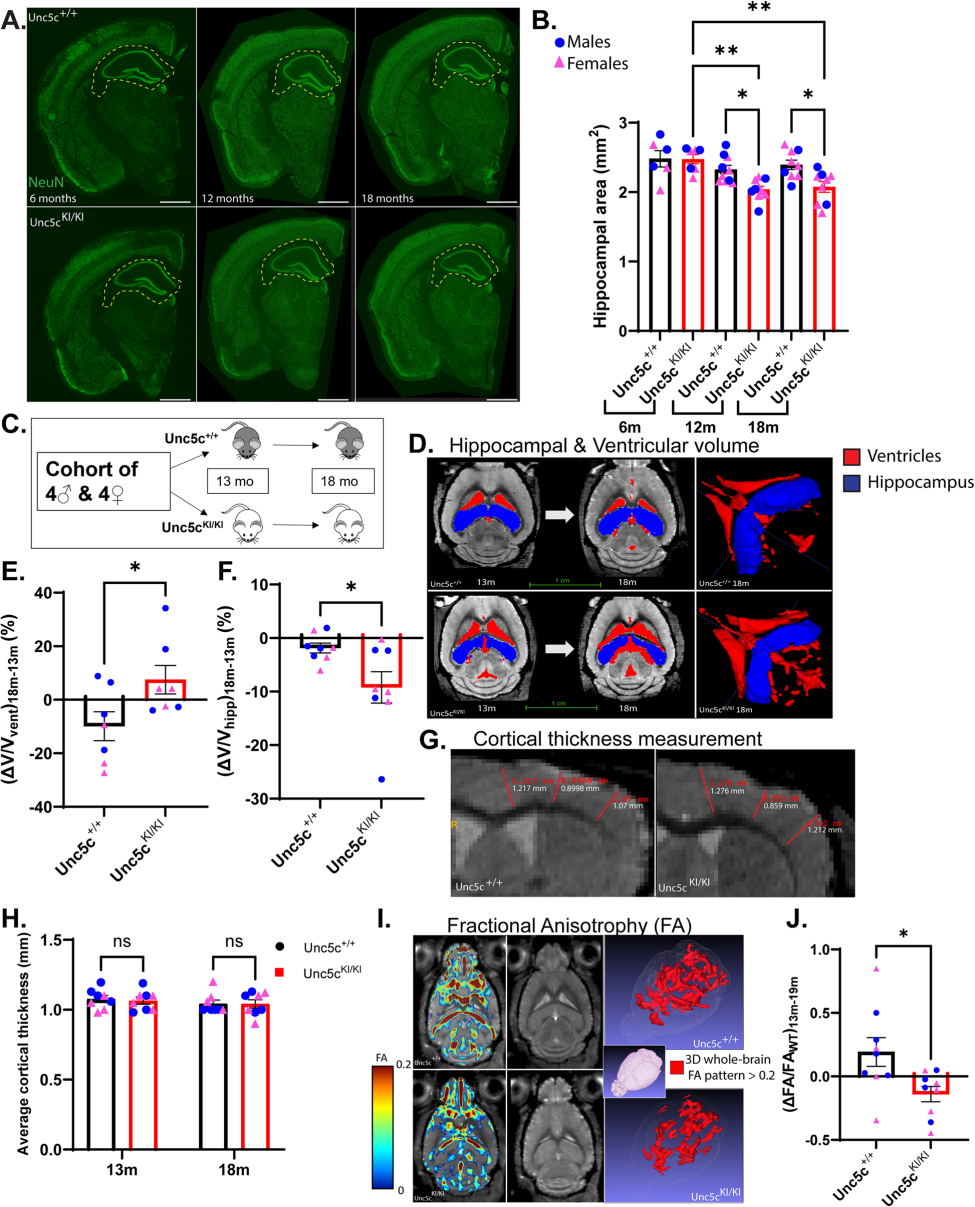
*Unc5c*^*KI/KI*^ mice show significant hippocampal atrophy with age. **A**. Coronal sections of hemibrains immunostained for NeuN (green) in *Unc5c*^+*/*+^ and *Unc5c*^*KI/KI*^ mice at 6, 12, and 18 months. Scale bar, 1.16 mm. Three different bregma positions (−1.34 mm (anterior), −1.70 mm (center), −2.06 mm (posterior)) were chosen for each animal to do the area analysis. Hippocampus is highlighted by dashed yellow region. **B**. Quantification of hippocampal area by ImageJ from 6–18 months. Blue circles—males; pink triangle—females. *n* = 4 females, 2 males (6 months), *n* = 5 females, 4 males (12 months), *n* = 6 females, 2 males (18 months). **C**. Schematic representation of the longitudinal MRI study. *n* = 8/genotype (4 males, 4 females) **D**. (Left) MRI slices with superimposed segmented regions of interest (Hippocampus (Blue), ventricle (red)) visualizing the changes in ventricle and hippocampus size over the course of ~ 5–6 months (13 to 18 month) for *Unc5c*^+*/*+^ (top) and *Unc5c*^*KI/KI*^ (bottom) mice. (Right) Representative 3D rendered MR images are shown for both *Unc5c*^+*/*+^ and *Unc5c*^*KI/KI*^ mice at 18 months. **E–F**. Quantification of the change over time (13 months to 18 months) in ventricular volume (**E**) and hippocampal volume (**F**). **G.** Representative MRI image slice showing the cortical thickness measurement. Red lines indicate various positions where the thickness was measured. Measurements (in white) in three different regions are similar in both *Unc5c*^+*/*+^ and *Unc5c*^*KI/KI*^ mice at 18 months. **H**. Quantification of cortical thickness in *Unc5c*^+*/*+^ and *Unc5c*^*KI/KI*^ mice at 13 and 18 months. **I**. Representative 2D MRI slices depicting the brains of *Unc5c*^+*/*+^ and *Unc5c*.^*KI/KI*^ mice with superimposed fractional anisotropy (FA) patterns thresholded at ~ 0.2 (red) across the whole brain. **J**. Quantification of the change over time (∆) in FA/FA_WT_ (13 months to 18 months). Statistics calculated using two-tailed unpaired student’s t-tests and ordinary one-way ANOVA using Tukey’s multiple comparison tests with Bartlett’s test correction. Data are presented as mean ± SEM. ns = non-significant. *p-value ≤ 0.05, ** *p*-value ≤ 0.01, *** *p*-value ≤ 0.001, and ****p-value of ≤ 0.0001

Assessment of cortical changes was done through linear measurements of cortical thickness using the built-in image annotation and linear measurement tool included in ITK SNAP. Measurements were done on the re-aligned high resolution 3D brain images at same exact anatomical location (prefrontal cortical area) for all subjects and at two symmetric locations ~ 2 mm off the brain's midline and ~ 1 mm in front of bregma (as shown in representative MRI image in Fig. 1M). Choice of using average of two contralateral measurements was done to reduce subjective choice of position and uncertainties in realignment. Linear measurements were selected for cortical assessment instead of volumetric analysis to avoid uncertainties linked to confounding selection of total cortical region to be included in the assay.

### Fractional Anisotropy (FA) analysis

We identified an MRI derived biomarker (thresholded FA—> 0.25) which can provide a quantitative volumetric value reflective of connectivity patterns (the index comes from dividing each thresholded volume FA ~ 0.25 by the whole brain volume).

### Imaging quantification and analysis using ImageJ and NIS-elements

For hippocampal and cortical area measurement, we employed immunofluorescence on serial sections for each animal stained with NeuN antibody. 10× images were obtained using Nikon Ti2 widefield microscope in the Northwestern Feinberg School of Medicine imaging core facility. Using ImageJ software, polygon selection was used to draw the outline of hippocampi on all the sections. We selected three different bregma positions (−1.34 mm (anterior), −1.70 mm (center), −2.06 mm (posterior)) to obtain the area of hippocampi and cortices (9 mice (5 females, 4 males) in *Unc5c*^*+/+*^*-Unc5c*^*KI/KI*^ analysis, 10 mice (5 females, 5 males) in NLGF-dKI analysis). The average of three positions were compared between *Unc5c*^*KI/KI*^ and *Unc5c*^*+*^*/*+ mice. For signal intensity measurement, region of interest was outlined using polygon selection in ImageJ, then the mean intensity was obtained.

For counting cells as in counting TUNEL + cells in the hippocampus, CD68 + cells, and both CD68 + and C1q + cells in microglia, we used the multi-point tool to count the cells manually in the stitched 40 × and 20 × hippocampal images, respectively, by someone blind to the genotypes of the animals. The images were obtained using AXR laser scanning confocal microscope (Northwestern University Nikon Imaging Centre) and the maximum projected images were used for quantification.

For the image analysis and quantification using Nikon NIS-Elements Software (Northwestern University Nikon Imaging Centre), recipes were created by setting intensity, size and background threshold for each staining to be quantified for the numbers or area covered (for GFAP and NeuN) in the region of interest (ROI). Once ROIs were drawn in cortex and hippocampus, a binary channel was created to run the recipe for each region. 10 × images obtained using a Ti2 wide-field microscope were used in these analyses. For plaque analysis and NeuN analysis in dKI and NLGF mice, the average of 3–5 sections from Bregma coordinates of about − 1.30 to − 2.52 mm was obtained. Recipes were created to obtain the different plaque core sizes by binning them into different size thresholds. Section selection, tracing, and volume analysis were performed by someone blind to the genotypes of the animals.

### IMARIS image reconstruction

Astrocyte and Microglial morphology were analyzed using the IMARIS software (v9.1) by reconstructing the z-stacks of 60× confocal images obtained from A1R laser scanning confocal microscope at the Nikon imaging facility at Northwestern university. For astrocyte 3D reconstruction, confocal z-stacks were imported into IMARIS. Maximum projection of confocal images were used to define GFAP (for astrocytes) signal for each image. Two sections per animal (*n* = 7–8 females, 3–5 males/genotype for *Unc5c*^+*/*+^ and *Unc5c*^*KI/KI*^ mice, *n* = 5 females, 5 males/genotype for NLGF and dKI mice) were used and the average was used for each animal. 18–22 cells/animal were used for the analysis and the average was calculated. Using the surface tool, GFAP channel was chosen. Using the filaments tool, the IMARIS software used slice rendering and calculated mean process length, area, volume, soma volume, number of processes, number of process branch points, and number of process terminal points. For analyzing TUNEL signal (red) within GFAP + astrocytes, we employed “section” tool to obtain the orthogonal view of a single plane to show that GFAP + astrocytes contained TUNEL signal.

### Synaptic dendritic orientation analysis

Maximum intensity projected 60 × images with PSD95 staining were analyzed using the OrientationJ plugin in ImageJ software by selecting the OrientationJ distribution and setting the local window σ to 3 pixels and gradient to “gaussian” [21]. Images were obtained with the CA1 neuronal layer and were rotated 90° to right or left. The pixel orientation distribution is displayed as histogram of gaussian window ranging from −90 to + 90°. Pixels that are parallel to the vector field (CA1 layer) are closer to 0° (parallel to the direction of dendritic processes emerging from the CA1 neuronal layer). As the dendrites digress from the parallel orientation, we checked for the degree of deviation in the KI mice. The resulting graph is presented as a Gaussian curve and the degree of deviation is represented by more distribution of orientation towards the ends of the curve (−89.5° and 89.5°).

### Tandem-mass tagged based Mass spectrometry (TMT-MS) sample preparation

TMT-MS sample preparation was performed as previously described [22]. Briefly, 200 μg homogenized hippocampal brain extracts were extracted using methanol-chloroform precipitation. The extracted protein was then resuspended in 6 M guanidine in 100 mM triethylammonium bicarbonate (TEAB) buffer (Thermo Scientific, Cat# 90,114). Subsequently, reduction and alkylation at Cysteine residues of proteins were performed by subsequent incubation with 5 mM dithiothreitol (DTT) and alkylated at free SH groups of cysteine residues with 20 mM iodoacetamide (IAA). Proteins were first digested for 3 h at room temperature (RT) with 1 μg of LysC (Promega, Cat# PI90307) and then overnight at 37 °C with 2 μg of Trypsin. The digest was then acidified with formic acid and desalted using C18 HyperSep columns (ThermoFisher Scientific, Cat# 60,108–302). The eluted peptide solution was dried before resuspension in 100 mM TEAB. Micro-BCA assay (Thermo Fisher Scientific, Cat#23,235) was subsequently performed to determine the concentration of peptides and 100 μg of peptides from each sample was then used for isobaric labeling. TMT 10-plex labeling was performed on peptide samples according to the manufacturer’s instructions (ThermoFisher Scientific). After incubating for 75 min at room temperature, the reaction was quenched with 0.3% (v/v) hydroxylamine. Isobaric labeled samples were then combined 1:1:1:1:1:1:1:1:1:1 and subsequently desalted with C18 HyperSep columns. The combined isobaric labeled peptide samples were fractionated into eight fractions using high pH reversed-phase columns (Thermo Fisher Scientific, Cat# PI84868). Peptide solutions were dried, and stored at − 80 °C.

### TMT-MS analysis

TMT-MS analysis was performed as previously described [22]. In short, samples were resuspended in 20μL of buffer A (5% acetonitrile, 0.125% formic acid), and micro-BCA was performed. 3 μg of each fraction was loaded for LC–MS analysis via an auto-sampler with a Thermo EASY nLC 100 UPLC pump onto a vented Pepmap100, 75 μm × 2 cm, nanoViper trap column coupled to a nanoViper analytical column (Thermo Scientific) with a stainless steel emitter tip assembled on the nanospray flex ion source with a spray voltage of 2000 V. Orbitrap Fusion was used to generate MS data. The chromatographic run was performed with a 4 h gradient beginning with 100% buffer A and 0% B and increased to 7% B over 5 min, then to 25% B over 160 min, 36% B over 40 min, 45% B over 10 min, 95% B over 10 min, and held at 95% B for 15 min before terminating the scan. Buffer A contained 5% acetonitrile (ACN) and 0.125% formic acid in H_2_O, and buffer B contained 99.875 ACN with 0.125% formic acid. Multinotch MS3 method was programmed with the following parameters: ion transfer tube temp = 300 °C, easy-IC internal mass calibration, default charge state = 2, and cycle time = 3 s. MS1 detector was set to orbitrap with 60 K resolution, wide quad isolation, mass range = normal, scan range = 300–1800 m/*z*, max injection time = 50 ms, AGC target = 6 × 10^5^, microscans = 1, RF lens = 60%, without source fragmentation, and datatype = positive and centroid [22]. Monoisotopic precursor selection was set to include charge states 2–7 and reject unassigned. Dynamic exclusion was allowed; *n* = 1 exclusion for 60 s with 10 ppm tolerance for high and low. The intensity threshold was set to 5 × 10^3^. Precursor selection decision = most intense, top speed, 3 s. MS2 settings include isolation window = 0.7, scan range = auto normal, collision energy = 35% CID, scan rate = turbo, max injection time = 50 ms, AGC target = 6 × 10^5^, and *Q* = 0.25. In MS3, the top 10 precursor peptides selected for analysis were then fragmented using 65% higher-energy collisional dissociation before orbitrap detection. A precursor selection range of 400–1200 m/*z* was chosen with mass range tolerance. An exclusion mass width was set to 18 ppm on the low and 5 ppm on the high. Isobaric tag loss exclusion was set to TMT reagent. Additional MS3 settings include an isolation window = 2, orbitrap resolution = 60 K, scan range = 120–500 m/*z*, AGC target = 6 × 10^5^, max injection time = 120 ms, microscans = 1, and datatype = profile.

### TMT-MS data analysis and quantification

TMT-MS data analysis was performed as previously described [22]. In short, protein identification, TMT quantification, and analysis were performed with The Integrated Proteomics Pipeline-IP2 (Integrated Proteomics Applications, Inc., http://www.integratedproteomics.com/). Proteomic results were analyzed with ProLuCID, DTASelect2, Census, and QuantCompare. MS1, MS2, and MS3 spectrum raw files were extracted using RawExtract 1.9.9 software (http://fields.scripps.edu/downloads.php) [23]. Pooled spectral files from all eight fractions for each sample were then searched against the Uniprot mouse protein database and matched to sequences using the ProLuCID/SEQUEST algorithm (ProLuCID ver. 3.1) with 50 ppm peptide mass tolerance for precursor ions and 600 ppm for fragment ions [24]. Fully and half-tryptic peptide candidates were included in the search space, all that fell within the mass tolerance window with no miscleavage constraint, assembled, and filtered with DTASelect2 (ver. 2.1.3) through the Integrated Proteomics Pipeline (IP2 v.5.0.1, Integrated Proteomics Applications, Inc., CA, USA). Static modifications at 57.02146 C and 229.1629 K were included [25]. The target-decoy strategy was used to verify peptide probabilities and false discovery ratios [26, 27]. A minimum peptide length of 6 was set for the process of each protein identification, and each dataset included a 1% FDR rate at the protein level based on the target-decoy strategy. Isobaric labeling analysis was established with Census 2 as previously described [28]. TMT channels were normalized by dividing it over the sum of all channels [27]. No intensity threshold was applied. The fold change was then calculated as the mean of the experimental group standardized values, and *p*-values were then calculated by Student’s *t*-test with Benjamini–Hochberg adjustment. Protein ontologies were determined with protein analysis through The **D**atabase for **A**nnotation, **V**isualization and **I**ntegrated **D**iscovery (DAVID). The gene-ontology term (GO term) obtained were sorted based on their Benjamini score. Protein ontologies with Fisher statistical tests with false discovery rate correction less than 0.05 were considered significant.

### Statistics

Statistics were calculated using Prism10 (GraphPad Software). Unpaired two-way Student’s t-test and ordinary one-way ANOVA using Tukey’s multiple comparison tests with Bartlett’s test correction were used in the data analysis. For the UNC5C blot, we performed both multiple t-test and two-way ANOVA with Sidak’s multiple comparisons test. Numbers of replicates and *P* values are stated in each figure legend. All data are plotted as means ± SEM. Significance was concluded when the *P* value was less than 0.05, indicated by *P < 0.05, **P < 0.01, ***P < 0.001. N.S. (not significant) denotes *P* > 0.05.

## Results

### *Hippocampal degeneration is evident in Unc5c*^*KI/KI*^* mice by 18 months of age*

The UNC5C T835M mutation is associated with LOAD [1], but the pathogenic mechanism of this variant in CNS neurodegeneration is unclear. To shed light on the pathophysiology of UNC5C T835M in AD, we used standard homologous recombination in ES cells to generate a targeted replacement mouse line that constitutively expresses this variant (Fig. S1A). We then analyzed the CNS phenotypes associated with UNC5C T835M expression in the targeted replacement mice, which were bred to homozygosity and aged up to 24 months. Using NeuN immunofluorescence microscopy, we found a significant reduction in hippocampal area in *Unc5c*^*KI/KI*^ mice compared to wildtype control *Unc5c*^+*/*+^ at 12 and 18 months compared to 6 months (Fig. 1A, B), while *Unc5c*^*KI/KI*^ and *Unc5c*^+*/*+^ cortical areas were equal at all ages, even up to 24 months of age (Fig. S1B, C). This observation may be related to higher expression levels of Unc5c in hippocampus compared to cortex [1]. We confirmed hippocampal volume loss in *Unc5c*^*KI/KI*^ mice in vivo over time using longitudinal magnetic resonance imaging (MRI) (Fig. 1C, D). We found that ventricular volume increased significantly between 13- and 18-months in *Unc5c*^*KI/KI*^ mice compared to *Unc5c*^+*/*+^ mice, (Fig. 1E, Fig.S1D) while hippocampal volume decreased significantly in *Unc5c*^*KI/KI*^ mice, both suggesting neurodegeneration (Fig. 1F, Fig.S1E). As in the immunofluorescence microscopy-based analysis, cortical thickness was equivalent in both *Unc5c*^*KI/KI*^ and *Unc5c*^+*/*+^ mice and did not change over time (Fig. 1G, H). We used diffusion tensor imaging (DTI) to measure the fractional anisotropy (FA) index, which is indicative of white matter connectivity. Between 13 and 18 months of age, *Unc5c*^*KI/KI*^ mice had a significantly greater decrease in FA than *Unc5c*^+*/*+^ mice, indicating reduced gray-matter interconnectivity in *Unc5c*^*KI/KI*^ mice (Fig. 1I, J).

### *Synaptic protein levels and dendritic organization are significantly altered in Unc5c*^*KI/KI*^* mice*

**Fig. 2:**
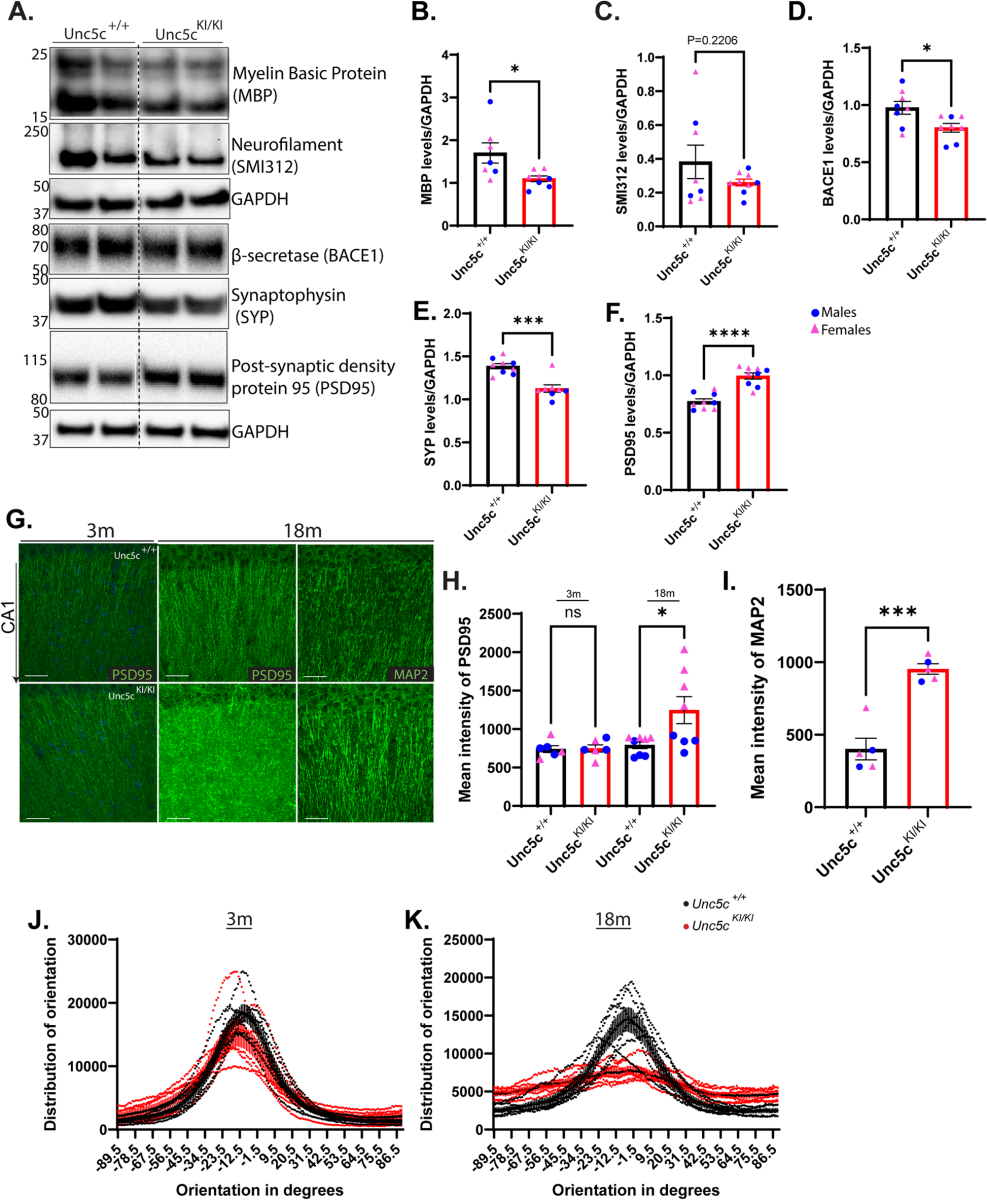
*Unc5c*^*KI/KI*^ mice have axonal and synaptic degeneration and dendritic disorganization** A**. Immunoblots of presynaptic/axonal and postsynaptic proteins in the hippocampal samples of 18-month-old *Unc5c*^+*/*+^ and *Unc5c*^*KI/KI*^ mice. **B**-**F**. Quantification of the immunoblots in (**A**) normalized to GAPDH. Blue circles—males; pink triangle – females. *n* = 4–5 females, 3–4 males/genotype. **G**. CA1 region of hippocampus stained for post-synaptic proteins such as PSD95 at 3-months and 18-months and MAP2 at 18 months in *Unc5c*^+*/*+^ and *Unc5c*^*KI/KI*^ mice. **H, I**. Quantification of mean fluorescence intensity of PSD95 (**H**) and MAP2 (**I**). **J, K** Graph showing the distribution of orientation of dendrites with respect to the CA1 nuclear layer obtained at 3 months (**J**) and 18 months (**K**) in *Unc5c*^+*/*+^ (black) and *Unc5c*^*KI/KI*^ (red) mice. *n* = 6 (*Unc5c*^+*/*+^), *n* = 7 (*Unc5c*.^*KI/KI*^). Statistics calculated using two-tailed unpaired student’s t-tests and ordinary one-way ANOVA using Tukey’s multiple comparison tests with Bartlett’s test correction. Data are presented as mean ± SEM. ns = non-significant. *p*-value ≤ 0.05, *p-value ≤ 0.01, **p-value ≤ 0.001, and ****p-value of ≤ 0.0001

Since the longitudinal MRI study strongly suggested white matter atrophy in the hippocampus, we hypothesized that the *Unc5c*^*KI/KI*^ mice had axonal and synaptic degeneration in the hippocampal region. Therefore, we assessed the levels of axonal and synaptic proteins in these mice. Immunoblot analysis of hippocampal homogenates of 18-month-old *Unc5c*^*KI/KI*^ mice appeared to have reduced presynaptic/axonal markers, Myelin basic protein (MBP), Neurofilament/pan-axonal marker (SMI312), β-secretase (BACE1), and synaptophysin (SYP) (Fig. 2A, B-E, Fig.S2). In contrast, immunoblots for the post-synaptic marker PSD95 surprisingly showed a significant increase in the *Unc5c*^*KI/KI*^ mice (Fig. 2A, F, Fig.S2). Post-synaptic changes were corroborated by immunofluorescence microscopy (Fig. 2G, H). Both MAP2 and PSD95 (post-synaptic markers) had increased immunostaining intensity in hippocampal CA1 (Fig. 2 G-I). Previous studies have shown that homeostatic synaptic plasticity exists to maintain the balance between pre- and post-synaptic sides of the synapse in the developing nervous system [29–31]. We hypothesized that if UNC5C T835M affects normal excitability or synaptic homeostasis, an abnormal decrease of the pre-synapse could cause a compensatory increase of the post-synapse. Another UNC protein, MUNC13, has also recently been implicated in controlling postsynaptic AMPA receptor density and clustering [32–34], suggesting a possible role for the UNC family, including UNC5 C, in the post-synaptic spine. Taken together, these data suggest that UNC5C T835M-mediated presynaptic degeneration is coupled with compensatory postsynaptic sprouting. Additionally, we observed that PSD95^+^ dendritic processes in the hippocampal CA1 region of the *Unc5c*^*KI/KI*^ mice showed abnormal organization compared to *Unc5c*^+*/*+^, which we measured as a loss of linearity in dendrites that run parallel to each other and perpendicular to the CA1 cell layer (Fig. 2J, K). When dendritic processes are parallel to each other, the angle between them is near 0°, as observed in 3-month-old mice and the 18-month-old *Unc5c*^+*/*+^ mice, but in 18-month *Unc5c*^*KI/KI*^ mice, the orientation is distributed nearly evenly, with no clear peak, indicating a loss of parallel organization. Even at 3 months in *Unc5c*^*KI/KI*^ mice, the peak near 0° is less pronounced and there are more values in the tails of the distribution, indicating that loss of linear organization may occur early and worsen with age. Taken together, our results show that the hippocampal atrophy exhibited by *Unc5c*^*KI/KI*^ mice is associated with reduced hippocampal presynaptic and axonal proteins, compensatory postsynaptic sprouting, and disorganized dendrites, suggestive of a neurodegenerative process.

### *Proteomic analysis reveals upregulation of oxidative stress and down-regulation of chaperone proteins in Unc5c*^*KI/KI*^* mice*

**Fig. 3:**
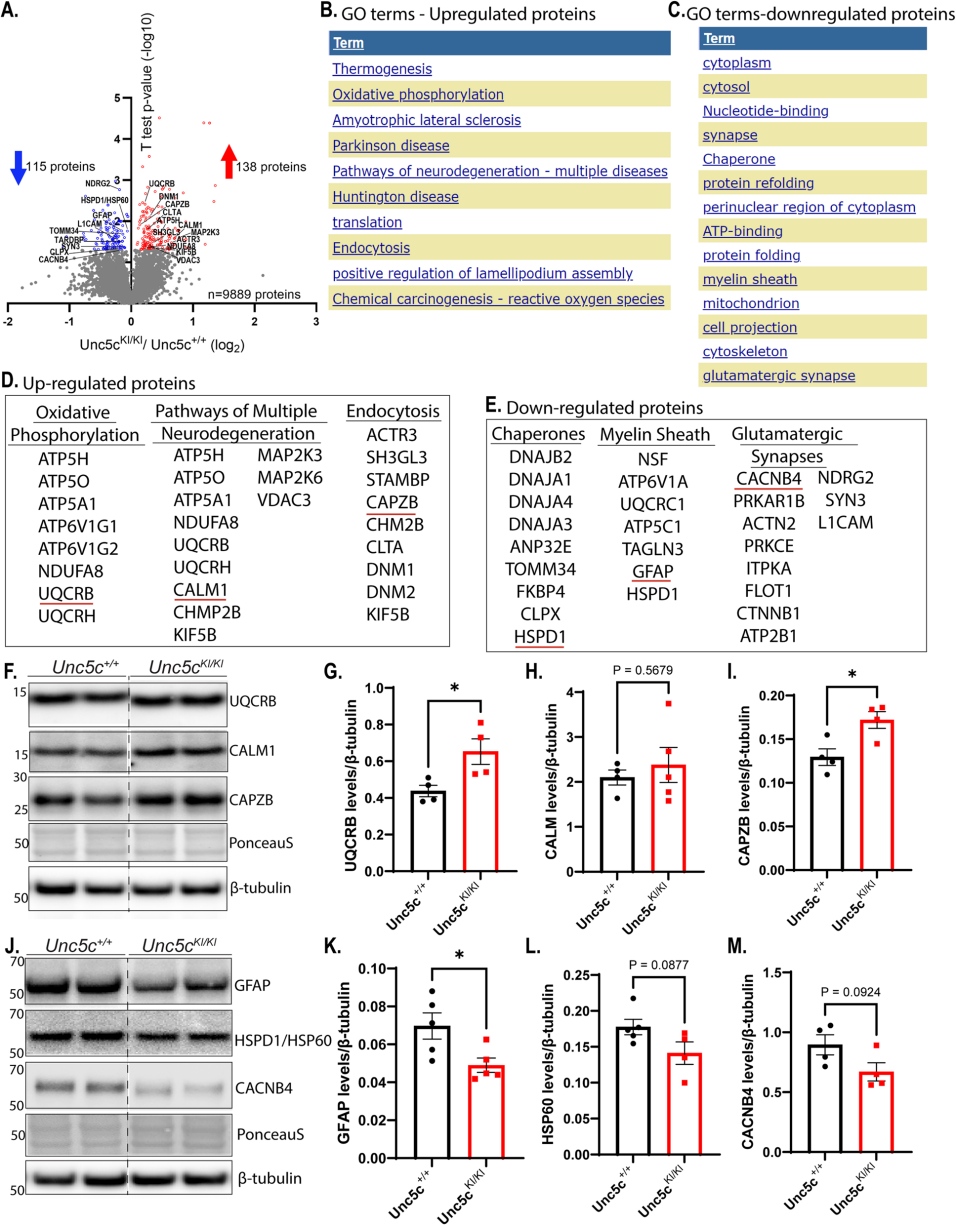
Proteomics reveal upregulation of oxidative stress and down-regulation of chaperone proteins in *Unc5c*^*KI/KI*^ mice hippocampi. **A**. Volcano plot of up-and down-regulated proteins obtained by TMT-MS of hippocampal homogenates of *Unc5c*^+*/*+^ and *Unc5c*^*KI/KI*^ mice at 18 months (*n* = 5 females/genotype). Number of up-regulated (red) and down-regulated proteins (blue) are shown along with total number of proteins obtained. **B**, **C**. DAVID analysis of up-regulated proteins (**B**) and down-regulated proteins (**C**) showing the most significant GO terms. **D, E**. List of proteins under each significantly up-regulated (**D**) and down-regulated (**E**) biological process/GO term in DAVID analysis. **F**. Immunoblot analysis of hippocampal homogenates from 18-month *Unc5c*^+*/*+^ and *Unc5c*^*KI/KI*^ mice for proteins (underlined in red in D) under each of the GO categories listed in D. **G**-**I**. Quantification of the immunoblots for UQCRB (**G**), CALM1 (H) and CAPZB (**I**) normalized to β-tubulin (*n* = 5 females/genotype). **J**. Immunoblot analysis of hippocampal homogenates from 18-month *Unc5c*^+*/*+^ and *Unc5c*.^*KI/KI*^ mice for proteins (underlined in red in E) under each of the GO categories listed in E. **K-M**. Quantification of the immunoblots for GFAP (**K**), HSPD1 (**L**) and CACNB4 (**M**) normalized to β-tubulin (*n* = 5 females/genotype). Statistics calculated using two-tailed unpaired student’s t-tests. Data are presented as mean ± SEM. ns = non-significant. *p*-value ≤ 0.05, **p-value ≤ 0.01, ***p-value ≤ 0.001, and ***p-value of ≤ 0.0001

We performed bulk proteomics on hippocampal extracts of *Unc5c*^+*/*+^ and *Unc5c*^*KI/KI*^ mice at 18 months to identify in an unbiased fashion the proteins that may reveal pathways and networks with important roles in UNC5C-mediated cell death (Fig. 3A, S8). We performed Gene Ontology:Biological processes (GO:BP) enrichment analysis for significantly upregulated proteins using **D**atabase for **A**nnotation, **V**isualization and **I**ntegrated **D**iscovery (DAVID) [35]. The terms that were most significantly upregulated included oxidative phosphorylation, Alzheimer’s disease, Parkinson’s disease, Huntington’s disease (pathways leading to neurodegeneration), and endocytosis (Fig. 3B, D, S8). To corroborate our proteomic results, we quantified by immunoblot the levels of specific upregulated proteins, including Calmodulin-1 (CALM1), ubiquinol-cytochrome c reductase binding protein (UQCRB), and Capping actin protein of muscle Z-line β subunit (CAPZB) that are involved in oxidative stress pathways leading to neurodegeneration and endocytosis (underlined in red in Fig. 3D, F, S8). Notably, UQCRB and CAPZB were significantly increased in *Unc5c*^*KI/KI*^ compared to *Unc5c*^+*/*+^ mice (Fig. 3G, I). Although CALM1 levels were not increased in the original *Unc5c*^*KI/KI*^ sample (Fig. 3H), normalization against PonceauS showed that CALM1 was significantly increased in *Unc5c*^*KI/KI*^ hippocampus (Fig.S3C). Remarkably, the down-regulated proteins by GO terms in the *Unc5c*^*KI/KI*^ mice were chaperone, myelin sheath, and glutamatergic synapse proteins (Fig. 3C, E). We validated the proteomic results with immunoblot analysis for some of the representative proteins from each term: heat shock protein family D (HSP60) member 1 (HSPD1/HSP60) (chaperone/protein folding), Glial fibrillary acidic protein (GFAP) (myelin sheath) and Calcium voltage-gated channel auxiliary β subunit 4 (CACNB4) (glutamatergic synapses) (underlined in red in Fig. 3E, J). Notably, GFAP was significantly decreased in the *Unc5c*^*KI/KI*^ compared to the *Unc5c*^+*/*+^ mice, while HSP60 and CACNB4 levels trended towards decrease in the *Unc5c*^*KI/KI*^ mice (Fig. 3K-M, Fig.S3), which were significant upon normalization with PonceauS (Fig.S3 F, G). Previously, it has been shown that increased endocytic activity along with increased trafficking to endosomes could possibly generate Aβ that could contribute to amyloid pathology and accelerate AD [36]. Together, our findings suggest that UNC5C T835M may promote oxidative stress, which in the presence of a cytotoxic stressor, such as pathologic Aβ or tau (AD) or α-synuclein (Parkinson’s disease), may cause disease pathogenesis.

### *Unc5c*^*KI/KI*^* mice exhibit increased apoptosis*

**Fig. 4:**
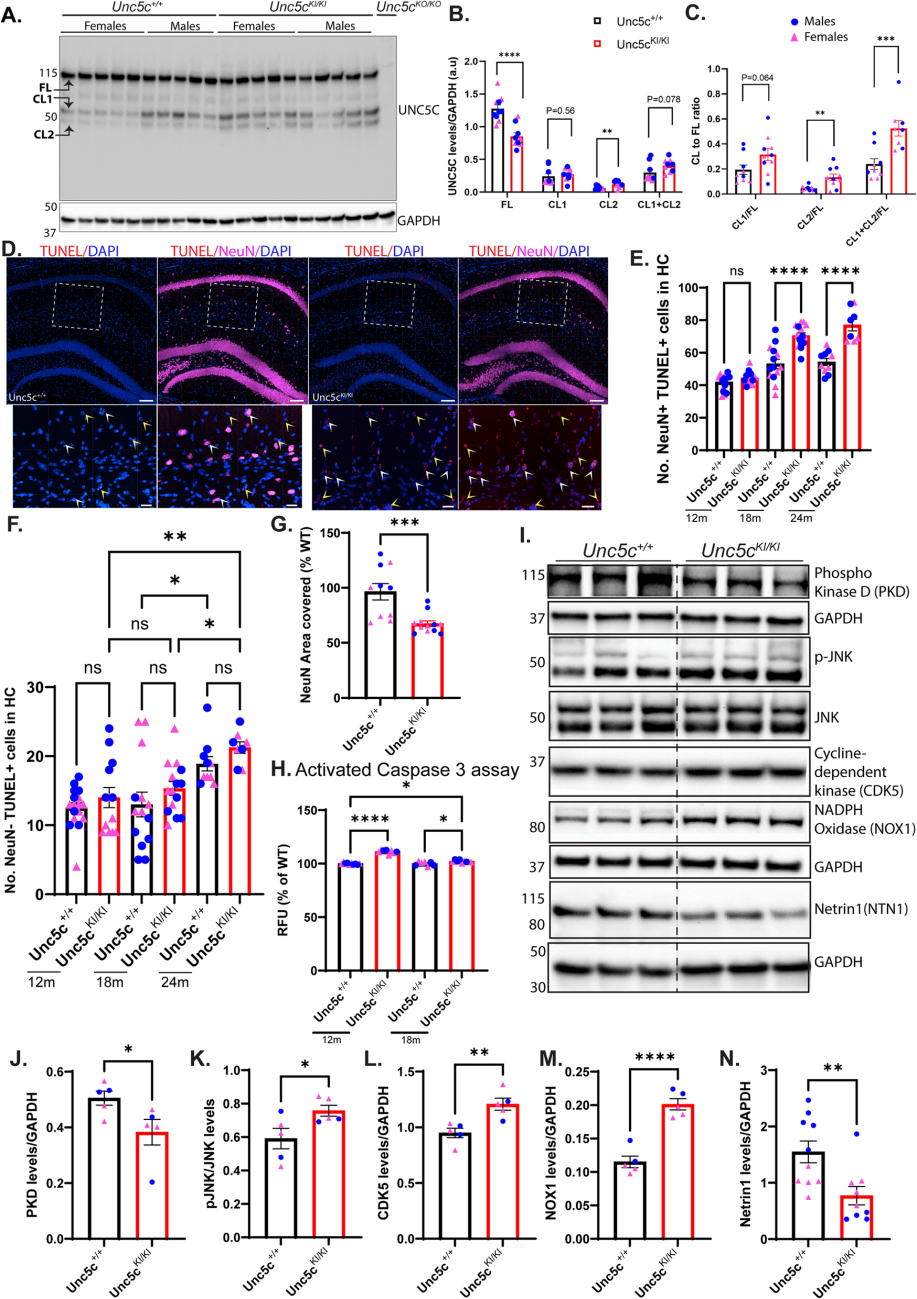
Increased neuronal apoptosis in *Unc5c*^*KI/KI*^ mice** A**. Immunoblot analysis using an UNC5C-specific antibody on 12-month-old hippocampal samples from *Unc5c*^+*/*+^ and *Unc5c*^*KI/KI*^ mice. *Unc5c*^*KO/KO*^ (*Unc5c*^*−/−*^) is used as a negative control. The Full-length (FL) UNC5 C band was observed around 115 kDa. Note two additional lower bands that are specific to the UNC5C antibody labeled Cleaved 1 (CL1) and Cleaved 2 (CL2) above and below the 50 k D marker, respectively. *N* = 10 (5 females, 5 males) **B**. Quantification of FL, CL1, CL2 and combined (CL1 + CL2) bands of UNC5C normalized to GAPDH and presented as arbitrary units (a.u). **C.** Quantification of the ratios of CL1, CL2, and combined CL1 + CL2 to FL bands. Blue circles – males; pink triangles – pink. **D.** Confocal images of CA1 region from 18m *Unc5c*^+*/*+^ and *Unc5c*^*KI/KI*^ mice stained for TUNEL-positive neurons (NeuN, magenta; TUNEL^+^, red). Scale bar, 100 μm. Sections around −1.70 mm Bregma position were chosen for analysis. White boxed region in upper panels is enlarged in lower panels. Yellow arrowheads show TUNEL^+^ cells, of which some are NeuN^+^ (white arrowheads). Scale bar, 20 μm. **E, F.** Quantification of number of TUNEL^+^ neurons (TUNEL^+^ NeuN^+^) (E) and non-neuronal TUNEL^+^ cells (TUNEL^+^ NeuN^−^) (F) in hippocampal sections of *Unc5c*^+*/*+^ (black) and *Unc5c*^*KI/KI*^ (red) mice. Blue circles—males; pink triangle—females. *N* = 5–7 males, *n* = 5–8 females/genotype/age. **G.** Quantification of the %NeuN covered area in the hippocampus at 18 months. *n* = 6 mice/genotype (2 sections/animal). **H.** Quantification of caspase-3 activity assay expressed as relative fluorescent units. *n* = 9–10 mice/genotype **I.** Immunoblot analysis of hippocampal homogenates from *Unc5c*^+*/*+^ and *Unc5c*.^*KI/KI*^ mice for proteins involved in UNC5 C T835M-mediated apoptosis pathway at 12 months. **J-N.** Quantification of immunoblot signals for Protein kinase-D (PKD) (**J**), phospho-JNK/JNK (**K**), cycline-dependent kinase (CDK5) (**L**), NADPH oxidase (NOX1) (**M**), Netrin1 (NTN1) (**N**) normalized to GAPDH. Blue circles—males; pink triangle—females. *n* = 3–5 females, *n* = 2–5 males/genotype. Statistics calculated using two-tailed unpaired student’s t-tests, multiple t-test, two-way ANOVA using Sidak’s multiple comparisons test (for panels B and C) and ordinary one-way ANOVA using Tukey’s multiple comparison tests with Bartlett’s test correction. Data are presented as mean ± SEM. ns = non-significant. *p-value ≤ 0.05, **p-value ≤ 0.01, ***p-value ≤ 0.001, and ****p-value of ≤ 0.0001

Since it was shown previously that UNC5C T835M increases susceptibility to death of hippocampal primary neurons [1], we employed the bioinformatic tool Polyphen-2 software (http://genetics.bwh.harvard.edu/pph2/) [37] to assess the impact of the T835M mutation on the structure and function of UNC5C protein. Polyphen-2 showed that the T835M substitution was “possibly damaging” to UNC5C protein structure with a score of 0.929 out of 1.0 (a mutation with a score of 0.0 is “tolerated” while that with 1.0 is “deleterious”; Fig. S4A), suggesting potential functional consequences that may explain the previously reported findings [1] and our own results of hippocampal atrophy suggesting neurodegeneration, as the mutation is in/near the hinge region and could affect the UNC5C open/closed state that triggers cell death or growth activities. Since cleavage of the intracellular domain of UNC5C and other UNC5 family members affect cell death via the apoptotic pathway [38–40], we generated an antibody against the C-terminal 400 amino acids of UNC5 C to assess how the T835M mutation affects protein levels and proteolytic processing in the hippocampus. We hypothesized that since the mutation is in the hinge region, it could cause a change in protein conformation favoring the ‘open’ state, thereby exposing the death domain that is susceptible to cleavage by activated caspase-3, triggering the apoptotic cascade. We observed that full length (FL) UNC5C levels were significantly reduced in *Unc5c*^*KI/KI*^ hippocampal homogenates by immunoblot analysis (Fig. 4A, B, Fig.S4B). Additionally, we observed two bands at ~ 57 kD and 52 kD – denoted as “CL1 and CL2”, respectively, corresponding to the expected cleaved products of UNC5C with activated caspase-3 (boxed regions, Fig. S4C), in *Unc5c*^+*/*+^ and *Unc5c*^*KI/KI*^ mice, but that were absent in *Unc5c* constitutive knockout mouse hippocampal homogenates (*Unc5c*^*KO/KO*^*)* (Fig. 4A, B, Fig.S4B). Both CL1 and CL2 levels were increased in *Unc5c*^*KI/KI*^ mice, although CL1 did not reach statistical significance (Fig. 4A, B). Importantly, the individual and summed ratios of CL1 and CL2 to full-length UNC5C were elevated in *Unc5c*^*KI/KI*^ mice, with the increase in CL2 being the main driver of the change (Fig. 4C). This observation, that signal for FL band is decreased while those of CL bands are increased, suggests a ‘precursor—product’ relationship. These results suggests that the T835M mutation might increase the cleavage of the FL UNC5 C protein into fragments that once released could in turn trigger the apoptotic cascade downstream of UNC5C when it is in its open conformation.

To support this hypothesis, we measured apoptotic cell death in *Unc5c*^+*/*+^ and *Unc5c*^*KI/KI*^ brains. Using a standard TUNEL (Terminal deoxynucleotidyl transferase (TdT) dUTP Nick-End Labeling) assay, we observed a significant increase in TUNEL + cells (Fig. 4D, Fig.S4D), specifically, NeuN +/TUNEL + cells (~ 60–80 cells) in the hippocampus of *Unc5c*^*KI/KI*^ mice at 18 and 24 months (Fig. 4D, E). The number of NeuN-/TUNEL + cells, representing microglia, astrocytes, endothelial cells, oligodendrocytes, and other cells, were far fewer compared to NeuN +/TUNEL + cells (~ 10–20 cells), and remained unchanged in *Unc5c*^*KI/KI*^ mice compared to that in *Unc5c*^+*/*+^ mice, indicating that only neurons are more susceptible to apoptosis in the *Unc5c*^*KI/KI*^ mice (Fig. 4F, Fig. S4E, F). Co-staining with GFAP (astrocytes), and Iba1 (microglia) showed there were very few TUNEL + GFAP + and TUNEL + Iba1 + cells in both *Unc5c*^+*/*+^ and *Unc5c*^*KI/KI*^ hippocampi (Fig. S4E, F). Additionally, NeuN-covered area was decreased by ~ 31% in the hippocampus of *Unc5c*^*KI/KI*^ compared to *Unc5c*^+*/*+^ mice (Fig. 4G). The activity of caspase-3, an effector caspase in the apoptotic process, was increased in hippocampal homogenates from *Unc5c*^*KI/KI*^ compared to *Unc5c*^+*/*+^ mice at 12 and 18 months of age (Fig. 4H), confirming that the UNC5C T835M mutation increases the susceptibility of neurons to cell death via apoptosis with age.

To further understand the molecular pathway of UNC5C-mediated neuron death, we performed immunoblot analysis for certain kinases known to be involved in apoptosis. Previous studies have shown that Protein Kinase-D (PKD) decreases induction of apoptosis by modulating the c-Jun N-terminal Kinase (JNK) pathway and phosphorylation of c-Jun [41]. Additionally, PKD1 has been shown to play an anti-apoptotic role in protecting neuronal cells in early stages of oxidative stress [42] by modulating JNK phosphorylation and preventing apoptosis. Since PKD1 has been shown to play a protective role in oxidative stress [43, 44], decreased PKD1 levels could lead to JNK phosphorylation and induce apoptosis via NAPDH oxidase (NOX1), as previously reported [2]. NOX1 has been associated with activated caspases in AD brains [45, 46]. Therefore, reduction in PKD levels might activate the JNK via increased phosphorylation, which then leads to elevated NOX1 levels. Consistent with increased apoptosis, we found a significant decrease in PKD in the hippocampus of *Unc5c*^*KI/KI*^ mice (Fig. 4I, J, Fig.S4G) as well as increased phosphorylation of JNK/SAPK, which has been shown to act downstream of UNC5C T835M [2] (Fig. 4I, K, Fig.S4G). Additionally, we observed increased Cyclin-Dependent Kinase 5 (CDK5) levels in *Unc5c*^*KI/KI*^ hippocampus (Fig. 4I, L, Fig.S4G), which have been implicated in apoptosis [47, 48]. Another study has shown that CDK5 induces c-Jun phosphorylation through activation of JNK by promoting oxidative stress [49]. Further, NADPH oxidase (NOX1) levels were increased in *Unc5c*^*KI/KI*^ hippocampus (Fig. 4I, M, Fig.S4G), providing additional evidence of an oxidative stress environment with age in the *Unc5c*^*KI/KI*^ mice. Taken together, our results strongly suggest that UNC5C T835M increases susceptibility to hippocampal neuron loss by creating an oxidative stress environment that leads to death via an apoptotic mechanism in vivo*.*

We also analyzed Netrin1 levels, which were significantly reduced at 12 months of age in the hippocampal samples from *Unc5c*^*KI/KI*^ compared to *Unc5c*^+*/*+^ mice (Fig. 4I, N, Fig.S4H). Since UNC5C is a member of the “dependence” receptor family, reduced Netrin1 (ligand) could initiate the apoptotic pathway [40]. This supports the hypothesis that the T835M mutation could cause a change in protein conformation that makes UNC5C more prone to adopting the “open” conformation when Netrin1 levels decrease, thus triggering apoptosis and neurodegeneration. Cytotoxic stressors such as Aβ could exacerbate this mechanism. Furthermore, reduced Netrin1 levels are also associated with increased amyloidogenic processing of APP [50], so the UNC5C mutation could have the dual effect of both inducing apoptotic neuronal death and driving amyloid pathology through Netrin1.

### *Reduced GFAP levels and morphological changes are observed in astrocytes of Unc5c*^*KI/KI*^* mice*

**Fig. 5:**
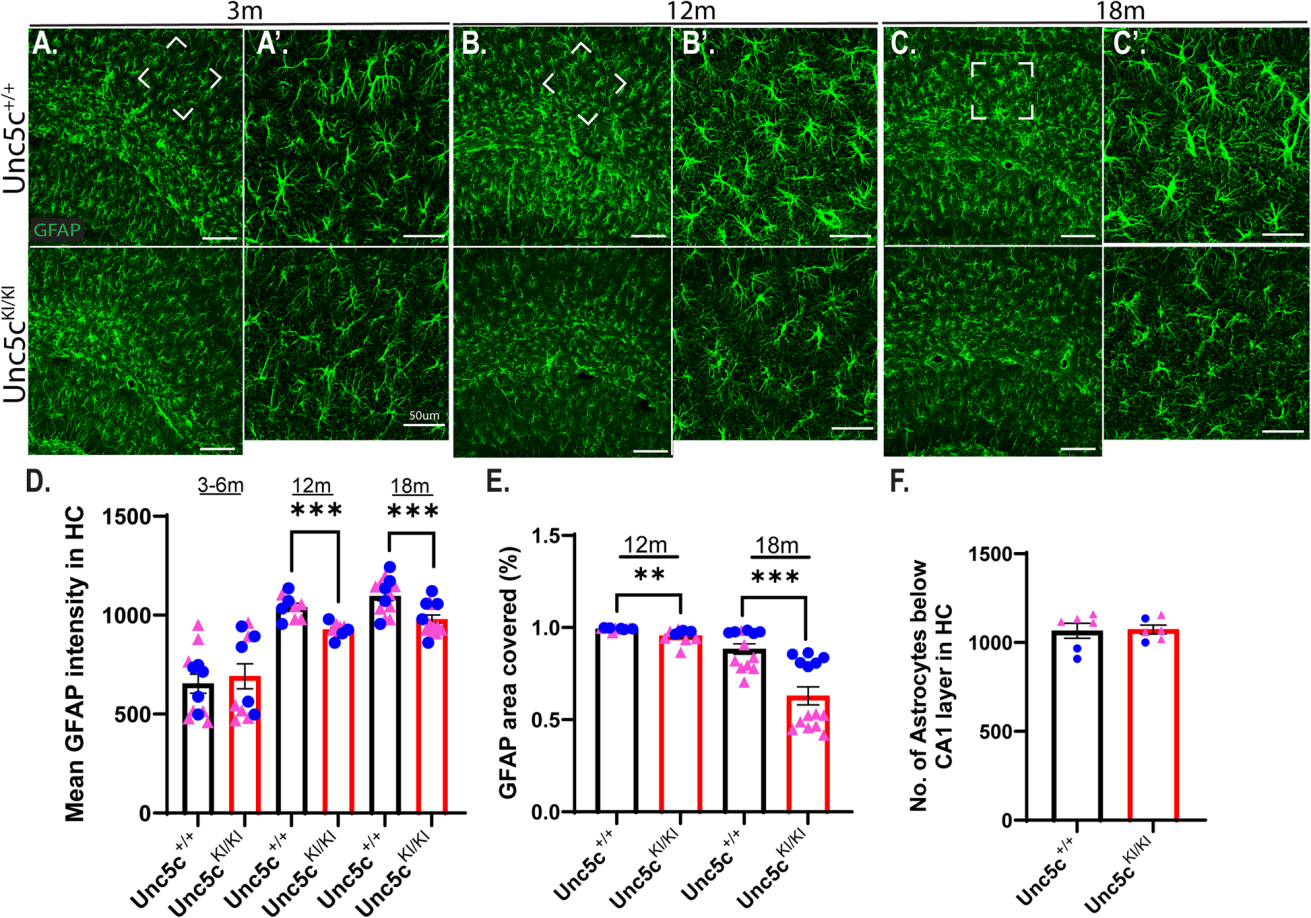
Astrocyte morphology is significantly altered in the *Unc5c*^*KI/KI*^ mice. **A-C**. Confocal images of CA1 region of *Unc5c*^+*/*+^ and *Unc5c*^*KI/KI*^ mice at 3 months (**A**), 12 months (**B**) and 18 months of age (**C**) immunostained for GFAP (astrocytes). Scale bar, 33 μm. Sections around −1.70 mm Bregma position were chosen for analysis. White-dashed boxed region is enlarged in **A’** (3 months), **B’** (12 months), and **C’** (18 months). Scale bar, 50 μm. **D.** Quantification of mean fluorescent intensity of GFAP in the CA1 region of hippocampus at 3–6 months, 12 months and 18 months in *Unc5c*^+*/*+^ and *Unc5c*.^*KI/KI*^ mice. **E.** Quantification of the % GFAP covered area in the hippocampus at 12 months and 18 months. Blue circles—males; pink triangle—females. *n* = 5–7 females, 5–7 males/genotype/age (2 sections/animal were used in the analysis). **F.** Quantification of number of astrocytes in the CA1 region of hippocampus at 18 months. *n* = 4 females, 3 males. Statistics calculated using two-tailed unpaired student’s t-tests and ordinary one-way ANOVA using Tukey’s multiple comparison tests with Bartlett’s test correction. Data are presented as mean ± SEM. Only comparisons with significant *p*-value are indicated. * *p*-value ≤ 0.05, ** *p*-value ≤ 0.01, ** *p*-value ≤ 0.001, and **** *p*-value of ≤ 0.0001

UNC5C is expressed in astrocytes as well as neurons [51] (https://brainrnaseq.org/?2327723709=1271088613) [52, 53]. Notably, we observed reduced GFAP levels in *Unc5c*^*KI/KI*^ hippocampal homogenates by proteomic analysis (Fig. 3J, K). Therefore, we used immunofluorescence microscopy to assess whether the UNC5C T835M mutation affected astrocytic phenotype (Fig. 5A-C). At 12 and 18 months, we observed a significant decrease in GFAP immunofluorescence (Fig. 5D) and GFAP + coverage area (Fig. 5E) in *Unc5c*^*KI/KI*^ mice, but no change in cell number (Fig. 5F) in the CA1 region of *Unc5c*^*KI/KI*^ mice. This suggested that astrocytes in *Unc5c*^*KI/KI*^ hippocampi are altered as a result of the mutation, since UNC5C is also expressed in astrocytes and our proteomics study showed that GFAP levels were down-regulated in the *Unc5c*^*KI/KI*^ mice (Fig. 3A, E). We speculate that UNC5C signaling may modulate GFAP expression in astrocytes, and that T835M may cause the observed GFAP reduction in *Unc5c*^*KI/KI*^ mice. GFAP comprises intermediate filaments of astrocytes and it has been shown that reduction/knockout of GFAP in astrocytes does not necessarily affect their survival [54]. Therefore, reduced GFAP levels could affect the cytoskeletal structure of astrocytes, which, in turn could affect their morphology. So, we next sought to examine the morphology of astrocytes in *Unc5c*^*KI/KI*^ mice. We used IMARIS software to perform 3D image reconstructions to measure astrocyte process length, volume, number of branch points, number of branch terminal points, number of dendrite terminal points, and soma area (Fig. S5A-M). Although *Unc5c*^*KI/KI*^ astrocytes exhibited more filamentous structure with increased branches, branch points and terminal points, we observed no change in the soma area of astrocytes (Fig. S5E). We speculate that astrocytes in *Unc5c*^*KI/KI*^ mice may compensate for neuronal cell death by branching out as a way of neuroprotection. Alternatively, the astrocytic changes could be playing a role in cell death, as decreased GFAP levels could be negatively affecting astrocyte functions such as providing cytoskeletal structure to astrocytes, as well as supporting neurons and endothelial cells in the neurovascular unit [55].

### *Microglia show increased activation in Unc5c*^*KI/KI*^* mice*

**Fig. 6:**
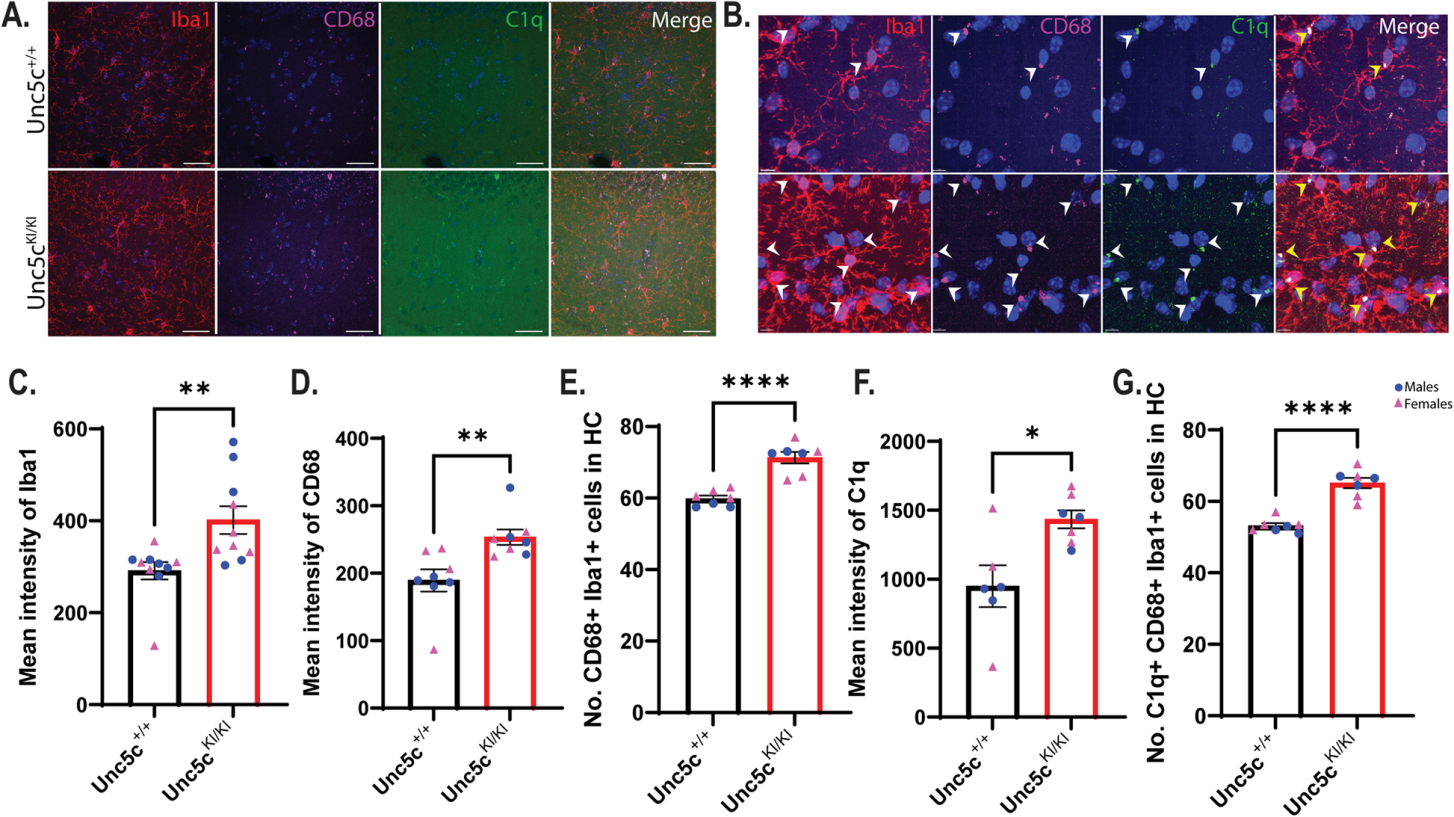
Microglia show increased activation in *Unc5c*^*KI/KI*^ mice. **A**. Confocal microscope images of CA1 of *Unc5c*^+*/*+^ and *Unc5c*^*KI/KI*^ mice at 18 months of age immunostained for Iba1 (red), CD68 (magenta) and C1q (green). Scale bar, 33 μm. **B**. Higher magnification of images in A showing increased activation of microglia (C1q^+^, CD68^+^) in the *Unc5c*^*KI/KI*^ mice. Scale bar, 7 μm. **C**, **D**, **F**. Quantification of mean fluorescence intensity of Iba1 (**C**), CD68 (**D**), and C1q (**F**) in *Unc5c*^+*/*+^ and *Unc5c*^*KI/KI*^ mice. **E, G**. Quantification of number of microglia (Iba1^+^) that are CD68^+^ (**E**) and both CD68^+^ and C1q.^+^ (**G**). Blue circles—males; pink triangle—females. *n* = 4–6 females, 3–5 males/genotype/age (2 sections/animal were used in the analysis). Statistics calculated using two-tailed unpaired student’s t-tests. Data are presented as mean ± SEM. Only comparisons with significant p-value are indicated. *p-value ≤ 0.05, ** *p*-value ≤ 0.01, *** *p*-value ≤ 0.001, and **** *p*-value of ≤ 0.0001

Although microglia do not express UNC5C under normal conditions, other members of UNC5 family can be upregulated under pathological/stress conditions in cultured microglia, AD mice and AD human brain [1, 56]. In addition, neuronal apoptosis and astrocytic changes caused by the UNC5C T835M mutation could affect microglia indirectly. To assess the effects of UNC5C T835M in microglia, we performed immunofluorescence microscopy, which revealed that *Unc5c*^*KI/KI*^ hippocampi had increased Iba1 and activated phagocytic microglial marker, CD68 compared to *Unc5c*^+*/*+^ mice at 18 months of age (Fig. 6A-E), demonstrating increased microglial activation. Additionally, the overall number of CD68 +/Iba1 + cells were significantly increased in *Unc5c*^*KI/KI*^ mice, supporting increased activated phagocytic microglia in the *Unc5c*^*KI/KI*^ mice (Fig. 6E).

Previous studies have shown that increased C1q expression in microglia correlated with increased synaptic engulfment and plays a role in neurodegeneration in an Alzheimer’s disease mouse model, and increased C1q levels were observed in hippocampi of patients with multiple sclerosis [57–59]. Further, excessive pruning of the excitatory synapses via complement-dependent pathway via C1q activation in microglia has been recently reported in AD mice [60]. As anticipated, we found increased C1q in *Unc5c*^*KI/KI*^ microglia by immunofluorescence microscopy, suggesting an elevation of complement-dependent synaptic engulfment by microglia in *Unc5c*^*KI/KI*^ mice (Fig. 6A, B, F, G). The Increase in Iba1, CD68 and C1q indicated activation of microglia in *Unc5c*^*KI/KI*^ mice, suggesting that microglia were reacting to degenerating neurons and possibly to astrocytic dysfunction (Fig. 6E, G). Alternatively, synaptic pruning by microglia could result from increased levels of PSD95 observed in *Unc5c*^*KI/KI*^ mice at 18 months to balance pre- and post-synaptic protein homeostasis (Fig. 2).

### UNC5C T835M-mediated neurodegeneration is exacerbated in dKI mice

**Fig. 7:**
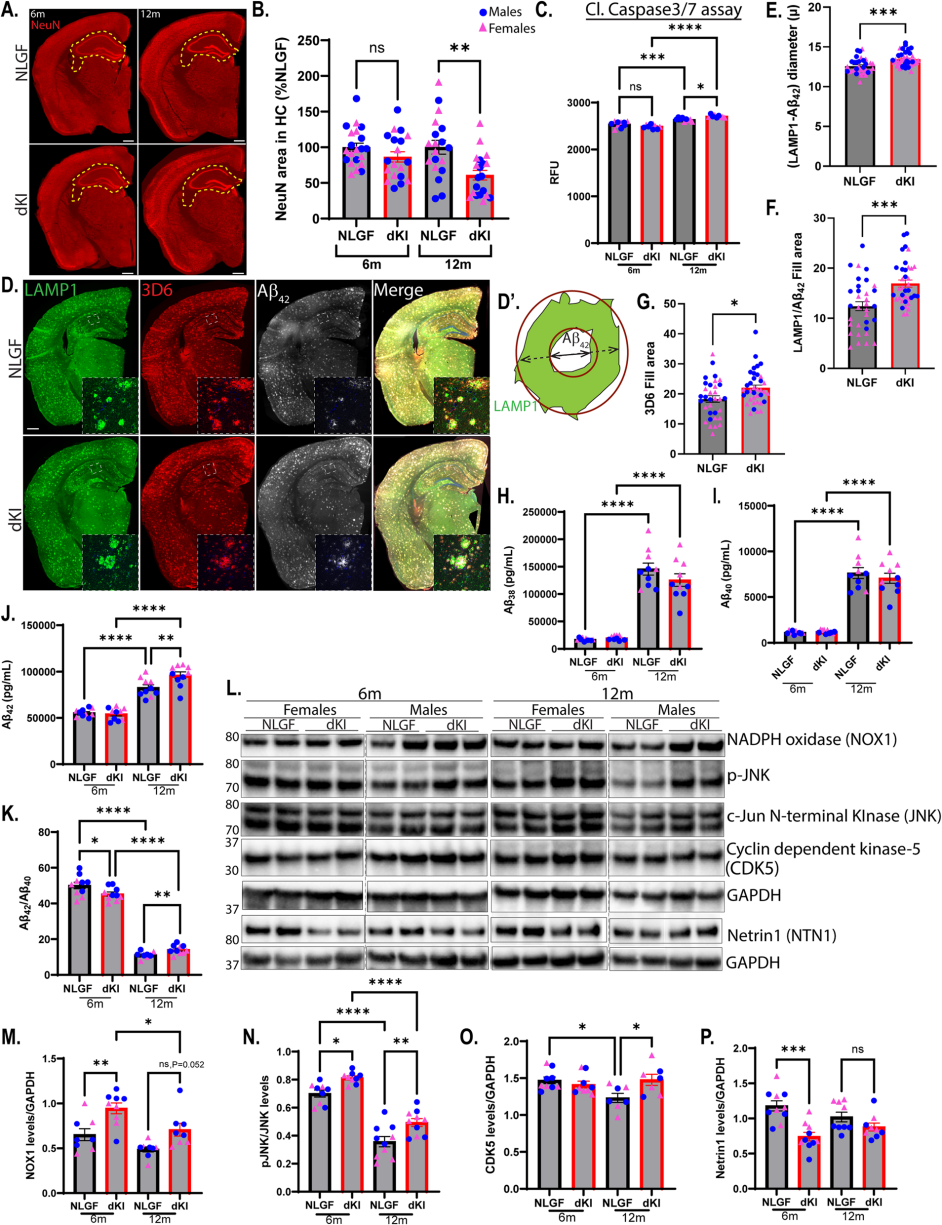
Neurodegeneration and UNC5C-mediated apoptosis are exacerbated in dKI mice. **A**. Confocal microscope images of CA1 of NLGF and dKI mice at 6 and12 months of age immunostained for NeuN (red). **B.** Quantification of the %NeuN covered area in the hippocampus at 6 and 12 months. *n* = 5 mice/genotype/age (2 sections/animal). **C**. Quantification of cleaved Caspase-3 activity assay expressed as relative fluorescent units in NLGF and dKI mice at 6 and 12 months. *n* = 9–10 mice/genotype/age. **D**. Coronal sections of hemibrains immunostained with LAMP1 (green), 3D6 (red), and Aβ_42_ (white) from NLGF and dKI mice at 12 months. Scale bar, 500 μm. Inset in each panel is a high-magnification image of the CA1 region outlined by a dashed box in the respective low-magnification image. **D’.** Outline of a plaque with the ‘halo’ marked by LAMP1 (green) and Aβ_42_ (white) to indicate how the diameter was measured. The diameter of the plaque core (Aβ_42_) is indicated by solid line while the dashed line is the diameter of LAMP1. **E–G.** diameter of the dystrophic neurites (LAMP1 – Aβ_42_ diameter) (**E**), Quantification of LAMP1/Aβ_42_ fill area (**F**), and 3D6 fill area (**G**) of NLGF and dKI mice at 12 months. *n* = 5 mice/genotype/age (3–5 sections/animal). **H–K.** MSD ELISA results of Aβ species – Aβ_38_ (**H**), Aβ_40_ (**I**), Aβ_42_ (**J**) and Aβ_42_/Aβ_40_ (**K**) of hippocampal homogenates from 6-month and 12-month-old mice. **L.** Immunoblot of hippocampal homogenates from NLGF and dKI mice for proteins involved in the UNC5C T835M pathway at 6 and 12 months. **M-P.** For immunoblots in L, quantification of NADPH oxidase (NOX1) (**M**), phospho-JNK/total JNK (**N**), cyclin-dependent kinase (CDK5) (**O**), Netrin1 (**P**) normalized to GAPDH. Blue circles—males; pink triangle—females. *n* = 5 mice/genotype/age. Statistics calculated using two-tailed unpaired student’s t-test and ordinary one-way ANOVA using Tukey’s multiple comparison tests with Bartlett’s test correction. Data are presented as mean ± SEM. Only data with significant *p* values are indicated. * *p*-value ≤ 0.05, ** *p*-value ≤ 0.01, *** *p*-value ≤ 0.001, and **** *p*-value of ≤ 0.0001

Since the UNC5C T835M mutation was shown to be associated with increased AD risk [1], we studied the UNC5C T835M phenotype in the context of an amyloid pathology to determine if it became worsened. Previous studies have shown that cytotoxic stressors such Aβ_42_, glutamate, and staurosporine exacerbate UNC5C T835M-mediated cell death in primary hippocampal neurons [1], so we hypothesized that T835M mutation would increase cell death in the *App*^*NL−G−F*^ mice (NLGF) mouse model, in which the APP gene is humanized with the Swedish double mutation (KM670,671 NL), as well as the Arctic (E693G) and Iberian (I716 F) mutations, that are all associated with autosomal dominant AD and promote amyloid pathology [16, 61]. We crossed *Unc5c*^*KI/KI*^ and NLGF mice to generate doubly homozygous NLGF;*Unc5c*^*KI/KI*^ (referred to as double KI, (dKI)) and NLGF;*Unc5c*^+*/*+^ (referred to as NLGF) mice. We observed a significant reduction in the neuronal area (NeuN stain) in dKI mice compared to NLGF mice at 12 months and a trend at 6 months, indicating increased hippocampal cell death in the dKI mice (Fig. 7A, B). To ascertain if there is increased cleaved caspase-3 activity, we measured active caspase-3 in hippocampal extracts from 6- and 12-month-old mice (Fig. 7C). Although there was no significant difference in caspase-3 activity between NLGF and dKI mice at 6 months, we observed a small but significant increase in caspase-3 activity in the dKI mice compared to NLGF mice at 12 months of age (Fig. 7C). Also, we observed a significant increase in caspase-3 activity in both genotypes at 12 months compared to 6 months, suggesting that NLGF mice alone do exhibit apoptotic cell death [62], which is significantly increased further in dKI mice (Fig. 7C).

Next, we determined whether amyloid pathology exacerbated UNC5C T835M-mediated effects on plaque-associated neuritic dystrophy, another indicator of neuronal dysfunction. To accomplish this, we measured the thickness of the LAMP1 positive halo, a commonly used marker of dystrophic neurites, around the Aβ_42_-defined plaque area [63] and found it to be increased in dKI mice (Fig. 7D, E). Another measure of neuritic dystrophy, the ratio of LAMP1:Aβ42, was also significantly increased in dKI mice (Fig. 7F), supporting increased neuronal/axonal damage and dysfunction in dKI compared to NLGF mice. We repeated these analyses with plaques binned by size and found that the dystrophic neurite increase observed in dKI was primarily driven by increased LAMP1 around smaller plaques under plaque core diameter of 20 µm (Fig. S6 A, B), which are thought to be most actively growing and most toxic to surrounding neuropil [64].

We then measured plaque coverage using a pan-Aβ antibody (3D6) and found it was increased in the hippocampal region of dKI mice compared to NLGF mice (Fig. 7D, G). To quantify each Aβ species, we performed MSD analysis to determine Aβ_38_, Aβ_40_, and Aβ_42_ levels in hippocampal homogenates (Fig. 7H-K). We observed that Aβ_42_ and Aβ_42_/Aβ_40_ ratio were increased at 12 months (Fig. 7J, K) while Aβ_38_ and Aβ_40_ levels remained unchanged (Fig. 7H, I), further supporting the amyloid-associated exacerbation of UNC5C T835M-induced neuronal degeneration in the presence of cytotoxic stressors [1]. We also used conventional ELISA kit to detect insoluble Aβ_42_ levels, and corroborated increased Aβ_42_ levels in dKI hippocampal lysates measured by MSD (Fig. 7J, Fig,S6 C).

We further wanted to understand how the molecular underpinnings of the UNC5C-mediated cell death pathway were affected in the presence of amyloid at 6- and 12-months. Since we observed an increase in p-JNK, CDK5 and NOX1 levels at 12 months in *Unc5c*^*KI/KI*^ mice, we expected that amyloid would exacerbate the UN5C-mediated apoptotic pathway. Indeed, we observed in the hippocampi of dKI mice a significant increase in NOX1 and phosphorylated JNK/SAPK/total JNK at 6 months (Fig. 7L-N, Fig.S6D, E), while CDK5 was significantly increased by 12 months (Fig. 7L, O, Fig.S6D, E), suggesting that the UNC5C T835M-mediated apoptotic pathway was exacerbated by amyloid pathology in dKI mice. Netrin1 (NTN1) has been shown to bind to APP and promote non-amyloidogenic processing of APP, thereby reducing the production of Aβ [65]. Conversely. reduced NTN1 correlates with increased Aβ in APP transgenic mice and human AD [9, 50, 66–68]. Therefore, NTN1 has been proposed as the therapeutic strategy for AD and may also reduce neuroinflammation [65, 68–73]. Furthermore, reduction of Netrin1 has been associated with Parkinson’s disease as well [50, 74–77]. We hypothesized that the reduced NTN1 levels in *Unc5c*^*KI/KI*^ mice (Fig. 4N) could lead to increased Aβ levels in dKI mice. Indeed, we not only observed increased Aβ_42_ levels and amyloid deposition in dKI mice (Fig. 7D, G, J), but also noted significantly decreased NTN1 levels in dKI compared to NLGF mice at 6 months, further supporting the hypothesis that the T835M mutation reduces Netrin1 levels, which in turn leads to increased Aβ in dKI mice (Fig. 7L, P, Fig.S6D, E).

### UNC5C T835M-mediated axonal degeneration/disorganization is exacerbated in dKI mice

**Fig. 8:**
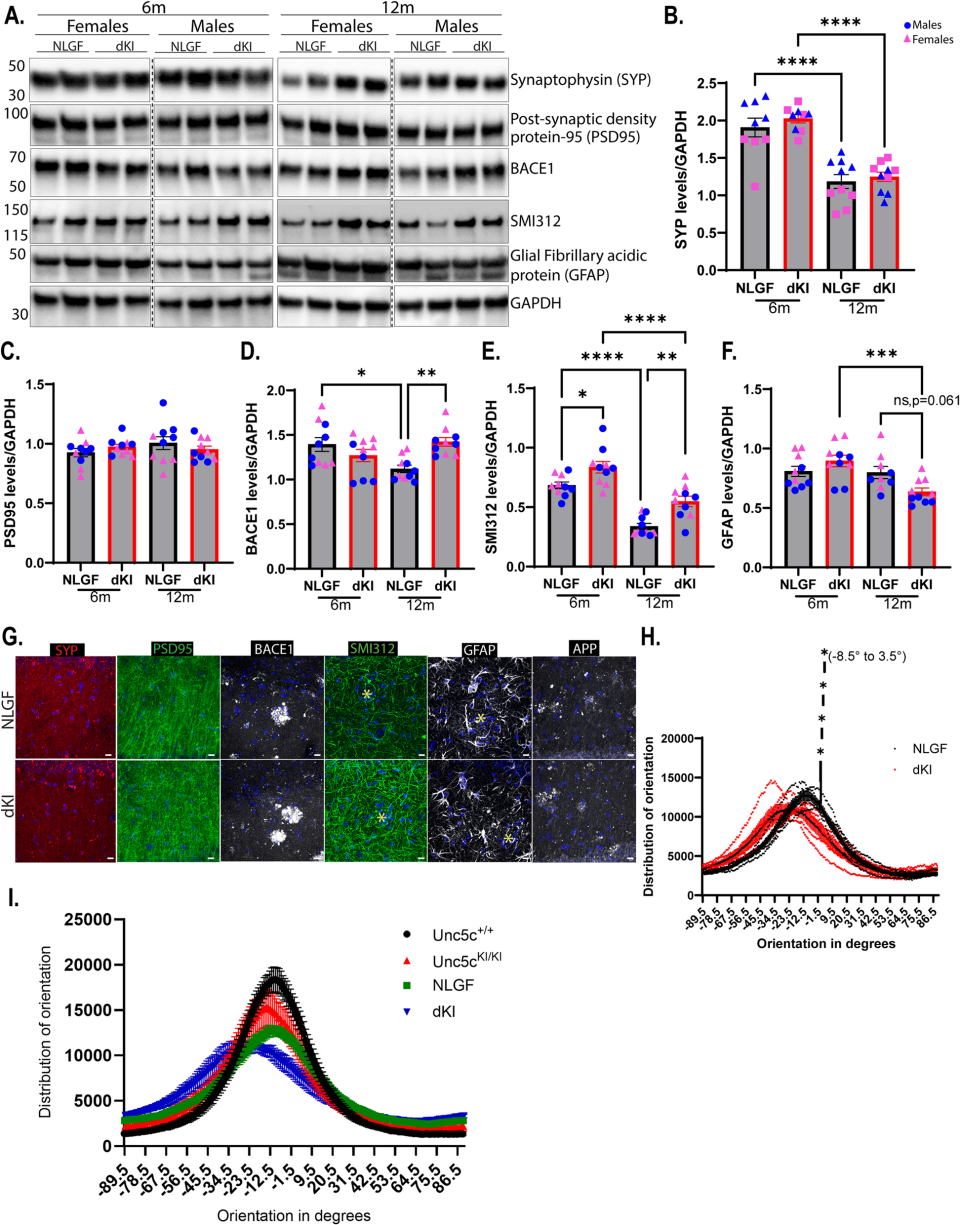
Synaptic abnormalities and dendritic disorganization are exacerbated in dKI mice**. A**. Immunoblot blot analysis of synaptic/axonal proteins in NLGF and dKI mice – PSD95, Synaptophysin (SYP), BACE1, GFAP and SMI312, at 6 and 12 months. *n* = 5/sex/genotype/age. **B-F.** Quantification of immunoblot signals for proteins in A normalized to GAPDH. **G.** Immunofluorescence microscopy for neuronal/synaptic proteins – BACE1, GFAP, SMI312 (phosphorylated neurofilament), PSD95, synaptophysin, and APP in NLGF and dKI mice at 12 months. Yellow star in panels with SMI312 and GFAP indicate the position of amyloid plaque. **H.** Graph showing the distribution of orientation of dendrites emerging from CA1 neuronal layer in NLGF and dKI mice at 12 months. There was a significant difference in the regions closer to 0° from (−8.5° to 3.5°) **I.** Overlay of the graph in **H** and graph in Fig. 2L showing dendritic orientation in NLGF and dKI mice at 12 months as compared to that of *Unc5c*^+*/*+^ and *Unc5c*.^*KI/KI*^ at 18 months as shown in Fig. 2L. Blue circles—males; pink triangle—females. *n* = 5/sex/genotype/age. Statistics calculated using two-tailed unpaired student’s t-test and ordinary one-way ANOVA using Tukey’s multiple comparison tests with Bartlett’s test correction. Data are presented as mean ± SEM. Only data with significant *p* values are indicated. *p-value ≤ 0.05, *p-value ≤ 0.01, *** *p*-value ≤ 0.001, and **** *p*-value of ≤ 0.0001

Since NLGF mice have synaptic loss by 6 months of age [16], and T835M *Unc5c*^*KI/KI*^ mice have a progressive loss of presynaptic markers with age, we hypothesized that the synaptic dysfunction in NLGF mice would be worsened by the UNC5C T835M mutation. Interestingly, by immunoblot analysis, synaptophysin, which is decreased in *Unc5c*^*KI/KI*^ compared to *Unc5c*^+/+^ was not different between NLGF and dKI hippocampi, although synaptophysin did decrease between 6 and 12 months in both genotypes (Fig. 8A, B, G, Fig.S7 A). Likewise, post-synaptic marker PSD95, which was increased in *Unc5c*^*KI/KI*^ compared to *Unc5c*^+*/*+^ was unchanged between NLGF and dKI mice (Fig. 8A, C, G, Fig.S7 A), suggesting that the synaptic changes of NLGF mask those of the UNC5C T835M mutation. Another presynaptic protein, β-secretase (BACE1), which was decreased in *Unc5c*^*KI/KI*^ compared to *Unc5c*^+*/*+^ mice (Fig. 2C) was significantly increased in dKI hippocampi at 12 months compared to NLGF (Fig. 8A, D, G, Fig.S7 A). This observation is likely due to increased dystrophic neurites in dKI mice as indicated by increased LAMP1:Aβ_42_ ratios (Fig. 7F), since BACE1 is well known to accumulate in dystrophic neurites [19, 78, 79] (Fig. 8A, D, G, Fig.S7 A). This is supported by the observation that neurofilament/pan-axonal marker (SMI312), which also accumulates in dystrophic neurite clusters surrounding plaques in AD brains [80] is increased in dKI mice Fig. 8A, E, G, Fig.S7 A). Overall, our results strongly suggest that dKI mice have increased dystrophic neurites with age, as measured by LAMP1, BACE1 and SMI312 immunolabeling and immunoblots (Fig. 7F, 8D, E, G, Fig.S7 A).

Interestingly, GFAP, which is usually increased in gliosis associated with amyloid, showed significantly decreased levels at 12 months in the dKI mice (Fig. 8A, F, G, Fig.S7 A), similar to what we observed in the hippocampi of *Unc5c*^*KI/KI*^ mice starting at 12 months. Further analysis of GFAP + astrocytes in NLGF and dKI mice showed that astrocytic morphology was not altered between the two genotypes, suggesting that plaques affect astrocytes similarly in both NLGF and dKI mice. However, only dKI mice showed significantly reduced GFAP levels (Fig. 8A, F, G, Fig. S7 A-J). Unlike the synaptic proteins, synaptophysin and PSD95, for GFAP, the effects of UNC5C T835M appear to over-ride the amyloid-associated phenotype typically observed in NLGF mice, although the astrocytic morphology remained unchanged. Finally, we measured the effect of UNC5C T835M on the degree of disorganization in CA1 dendrites. As before, we measured linearity in the dendritic processes that run parallel to each other and perpendicular to the CA1 cell layer. We observed that there was a significant difference near −10° to + 10° in the dKI mice, compared to NLGF, similar to the difference seen between *Unc5c*^*KI/KI*^ and *Unc5c*^+*/*+^ mice, suggesting that there is an exacerbation of the dendritic disorganization in the dKI mice compared to NLGF mice (Fig. 8H). When we compared the degree of disorganization between NLGF and dKI mice at 12 months (Fig. 8H) and *Unc5c*^+*/*+^ and *Unc5c*^*KI/KI*^ mice at 18 months (Fig. 2L), we found that the dendrites in the CA1 region of the hippocampus in dKI mice had the highest degree of disorganization compared to the other genotypes (Fig. 8I).

## Discussion

**Fig. 9:**
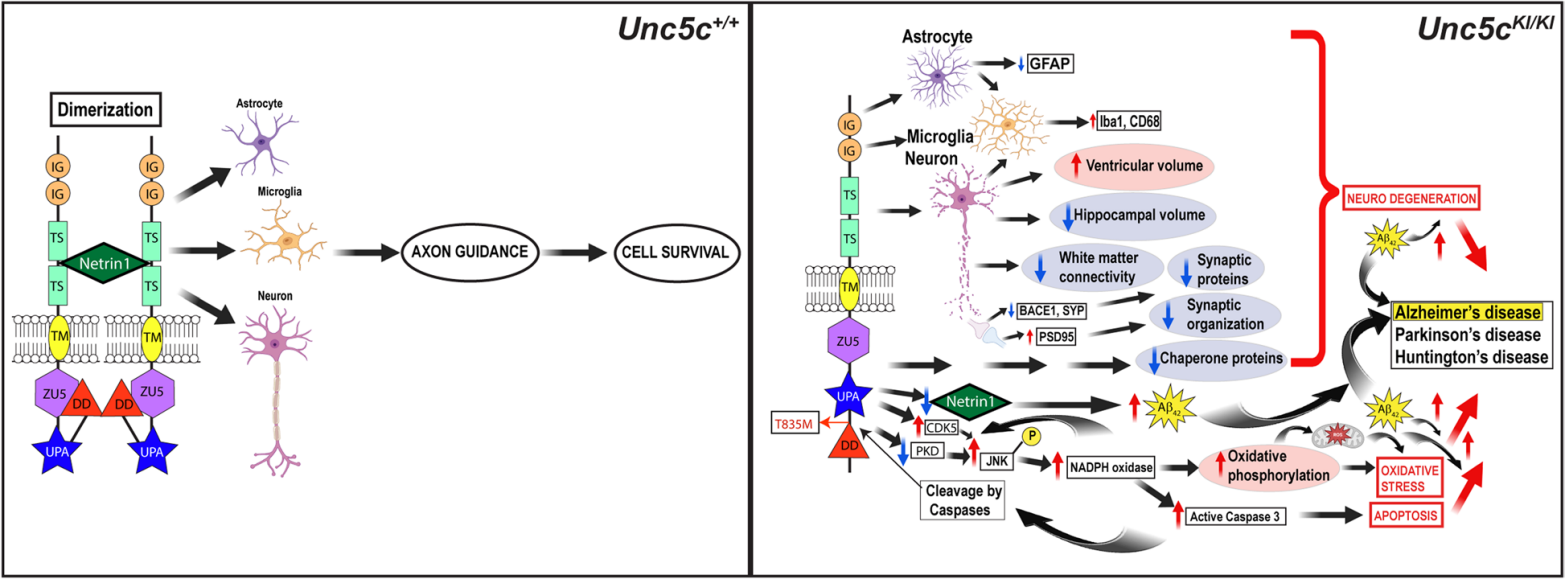
Mechanism of UNC5C T835M-mediated cell death and neurodegeneration. (Left) In WT mice (*Unc5c*^+*/*+^), UNC5C performs its function as dependence receptor in axon guidance pathway in the presence of its ligand Netrin1. (Right) UNC5C T835M (*Unc5c*^*KI/KI*^) mutation leads to neurodegeneration via synaptic degeneration, disorganization and apoptotic cell death in neurons, reduced GFAP levels and changed morphology with more processes in astrocytes and activated microglia. The mutation results in decreased and increased PKD and CDK5 levels, respectively, leading to activation of the JNK pathway, with further increase in NADPH oxidase (NOX1) creating an oxidative stress environment. With reduced chaperone proteins, NOX1 could trigger the activation of cleaved caspase-3 leading to apoptosis. The UNC5C T835M mutation also results in decreased Netrin1, which could elevate Aβ_42_ production, in addition to increased apoptosis. Overall, this pathway leads to increased neuronal loss, reduced hippocampal volume and increased ventricular volume in *Unc5c*^*KI/KI*^ mice. Thus, *Unc5c*^*KI/KI*^ mice provide an ideal environment for the study of various neurodegenerative diseases such as Alzheimer’s disease, Parkinson’s disease, or Huntington’s disease. UNC5C-mediated neurodegeneration, oxidative stress, and apoptosis are exacerbated in the presence of β-amyloid. Images created with BioRender.com

Here, we report the phenotypic characterization of a novel KI mouse model of the UNC5C T835M variant associated with LOAD, which makes the neurons more susceptible to stress-induced cell death, as previously reported by Wetzel Smith-Hunkapillar et al. [1]. UNC5C is necessary and essential for survival and maintenance of neurons and astrocytes during development and in aging by the protein dimerization and Netrin1 binding (Fig. 9, left). We hypothesize that UNC5C T835M could either introduce a kink in the protein conformation or reduce Netrin1 levels or both, which then could lead to biasing the protein to be in the open conformation leading to caspase-3 cleavage and activation of the downstream apoptotic cascade involving reduced Netrin1 and PKD levels, consequent JNK phosphorylation, and increased NADPH oxidase levels. NADPH oxidase activates additional caspases leading to apoptosis of neuronal cells. Although other modes of cell death such as necroptosis, ferroptosis, pyroptosis, and parthanatos in principle could be engaged with aging and neurodegenerative diseases such as in AD [81], UNC5 family members have been well established to be involved in the apoptotic pathway [14, 15, 38–40]. Therefore, we focused on whether or not the UNC5C T835M mutation increased apoptosis by investigating Capsase-3 activity and TUNEL signal in *Unc5c*^*KI/KI*^ brains. Our results showed that there is indeed a significant increase in caspase-3 activity and TUNEL + neurons, suggesting neuronal cell death via apoptosis (Fig. 4A-H). Taken together, our results strongly suggest that UNC5C T835M increases the open conformation leading to elevated caspase cleavage and apoptosis, as well as phosphorylation of JNK pathway through decreased and increased PKD and CDK5, respectively. Subsequently, NOX1 levels are increased causing an oxidative stress environment that in turn increases caspase-3 activity in a vicious cycle, resulting in hippocampal neuron death via apoptosis (Fig. 4J-M). Neuronal degeneration could affect astrocytes or astrocytic dysfunction to diminish neuronal health. However age, being the most important contributing factor for AD, in the context of UNC5C T835M may result in a significant reduction in hippocampal volume and increase in ventricular volume of the brain (Fig. 1E, F), reinforcing the association of UNC5C T835M with LOAD [1]. Further, the mutation may affect microglia indirectly because of chronic insults to neurons and astrocytes. *Unc5c* has been shown to be expressed at high levels in oligodendrocytes ( [52, 53]. Indeed, we observed significantly reduced levels of MBP (Fig. 2A, B) and our proteomics data showed that the GO term “myelin sheath” was down-regulated in *Unc5c*^*KI/KI*^ mice (Fig. 3C, E). These data suggest that the UNC5C T835M mutation could affect oligodendrocytes as well, which is a topic to be investigated in future studies. These individual cellular phenotypes combined appear to contribute to oxidative stress in the *Unc5c*^*KI/KI*^ mouse brain, providing an ideal environment for neurodegenerative diseases such as AD, PD, or Huntington’s disease (Fig. 9, right). Further, reduced Netrin1 levels have been shown to increase the amyloidogenic processing of APP leading to increased Aβ_42_ levels [71], which we show to be the case in dKI mice (Fig. 7D, G, J, P). The whole mechanism, including neurodegeneration, synaptic degeneration/disorganization and oxidative stress leading to apoptosis, is exacerbated in the presence of amyloid (dKI mice) resulting in neuronal cell death and AD pathogenesis (Fig. 9, right).

In this study, we employed *Unc5c*^*KI/KI*^ homozygous mice, although in human patients, the mutation typically presents in a heterozygous condition. Since the phenotypes in *Unc5c*^*KI/KI*^ mice exhibited as late as 12–18 months, we did not explore these phenotypes in *Unc5c*^*KI/*+^ mice, which we predict would present at even older ages. In humans, where LOAD occurs over the age of 70 and takes at least 2 decades to develop, a single UNC5C T835M mutant allele may be sufficient to increase susceptibility to AD pathogenesis. Behavioral studies were conducted on 9-month and 12-month-old *Unc5c*^+*/*+^ and *Unc5c*^*KI/KI*^ mice but did not yield any conclusive evidence of hippocampal memory impairment in the *Unc5c*^*KI/KI*^ mice (data not shown), probably because the neuronal loss just begins around that age causing behavioral deficits to manifest much later.

Overall, we report an age-associated loss of hippocampal volume and increased hippocampal neuronal loss with age in UNC5C T835M targeted replacement mice, which could serve as a model to study other neurodegenerative diseases such as Parkinson’s and Huntington’s disease. Future studies inhibiting NOX1 or blocking activation of UNC5C-cleaving caspases, or supplementing with Netrin1, to test their ability to ameliorate AD pathology and neurodegeneration, could potentially lead to new therapeutic agents for AD.

## Supplementary Information

Additional file 1: Supplementary figure S1: Hippocampal neurodegeneration is observed at 18 months in the *Unc5c*^*KI/KI*^ mice. A. Schematic representation of UNC5C T835M Exon 15 knock-in targeting vector B. Confocal image highlighting the cortical area with dashed yellow region at 24 months in *Unc5c*^*+/+*^ and *Unc5c*^*KI/KI*^mice. Scale bar, 1.16 mm. C. Quantification of cortical area by ImageJ at 24 months. *n*=7, *Unc5c*^*+/+*^; *n*=8, *Unc5c*^*KI/KI*^. Blue circles - males; pink triangle - females. D, E. Graph showing the change in volume of ventricles and hippocampus over time for individual animals used in the study. Statistics calculated using two-tailed unpaired student’s t-tests. Data are presented as mean ± SEM. Only comparisons with significant *p*-value are indicated. * *p*-value ≤ 0.05, ** *p*-value ≤ 0.01, *** *p*-value ≤ 0.001, and **** *p*-value of ≤ 0.0001.

Additional file 2: Supplementary figure S2: Uncut blots for the axonal/pre- and post-synaptic proteins and loading controls with red boxes showing the bands represented in Fig. 2 A. Blots were cut around the protein size to probe the protein of interest. Loading control for each blot used is shown underneath the proteins investigated.

Additional file 3: Supplementary figure S3: A. Uncut blots for the up-regulated and down-regulated proteins loading controls and PonceauS with red boxes showing the bands represented in Fig. 3D, J. Asterisk in GFAP and CACNB4 blots indicate non-specific bands and were not used in the analysis. B-G. Quantification of the immunoblots for UQCRB, CALM1and CAPZB, GFAP, HSPD1/HSP60 and CACNB4 normalized to PonceauS. Statistics calculated using two-tailed unpaired student’s t-tests. Data are presented as mean ± SEM. Only comparisons with significant *p*-value are indicated. * *p*-value ≤ 0.05, ** *p*-value ≤ 0.01, *** *p*-value ≤ 0.001, and **** *p*-value of ≤ 0.0001.

Additional file 4: Supplementary figure S4: Increased neuronal apoptosis in *Unc5c*^*KI/KI*^ mice. A. Snapshot of results of Polyphen-2 software showing the effects and score of the T835M mutation on UN5C protein structure and function. B. PonceauS stain showing the loading of *Unc5c+/+*, *Unc5c*^*KI/KI*^ and *Unc5c*^*KO/KO*^ hippocampal samples for the UNC5C blot in Fig. 4B. C. Pairwise alignment of the human and mouse UNC5C protein sequences with the predicted Caspase-3 cleavage sites in red boxes and the predicted sizes of the cleaved fragments to be obtained from those cleavage sites are indicated above those boxes. D. Quantification of number of TUNEL+ cells in hippocampal sections of *Unc5c*^*+/+*^ and *Unc5c*^*KI/KI*^ mice. Blue circles - males; pink triangle - females. *N*=5-7 males, *n*=5-8 females/genotype/age. Statistics calculated using two-tailed unpaired student’s t-tests and ordinary one-way ANOVA using Tukey’s multiple comparison tests with Bartlett’s test correction. E. A single-plane orthogonal view of a confocal image of CA1 region from 18 m *Unc5c*^*+/+*^ and *Unc5c*^*KI/KI*^ mice stained for TUNEL-positive cells. Scale bar, 20 μm, F. 20x confocal images of CA1 region from 18 m Unc5c+/+ and *Unc5c*^*KI/KI*^ mice stained for TUNEL-positive cells. Scale bar, 20 μm. G, H. Uncut blots for proteins involved in UNC5C T835M-mediated apoptosis, loading control and netrin1, loading control with red boxes showing the bands represented in Fig. 4H. Data are presented as mean ± SEM. Only comparisons with significant *p*-value are indicated. * *p*-value ≤ 0.05, ** *p*-value ≤ 0.01, *** *p*-value ≤ 0.001, and **** *p*-value of ≤ 0.0001.

Additional file 5: Supplementary figure S5: Astrocyte morphology is significantly changed in the *Unc5c*^*KI/KI*^ mice. A-D’. Step-wise IMARIS 3D reconstruction showing the morphology of astrocytes in the CA1 region of *Unc5c*^*+/+*^ and *Unc5c*^*KI/KI*^ mice. Scale bar, 15μm - The original image with astrocytes stained with GFAP and DAPI (A), Image rendered after applying the surface tool to show the astrocytic processes (B), Further slice rendering under the surface tool to fill the astrocytic processes (C), magnified image focusing on a single astrocyte highlighted in the dashed box in B (D), scale bar, 15μm, Filament tool reconstruction of astrocyte circled in D showing different analyzed parameters – soma, branch points, terminal points, dendrite branches, (D') scale bar, 7 μm. E-M. Quantification of astrocytic processes parameters including soma area, volume to area ratio, process length, area, volume, number of dendritic branches, number of branch terminal points, number of dendritic segmentsand dendritic terminal points. *n*=6-8 females, 5-7 males/genotype/timepoint. Statistics calculated using two-tailed unpaired student’s t-tests and ordinary one-way ANOVA using Tukey’s multiple comparison tests with Bartlett’s test correction. Data are presented as mean ± SEM. Only comparisons with significant *p*-value are indicated. * *p*-value ≤ 0.05, ** *p*-value ≤ 0.01, *** *p*-value ≤ 0.001, and **** *p*-value of ≤ 0.0001.

Additional file 6: Supplementary figure S6: Plaque associated dystrophic neurites are inversely proportional to the plaque core. A, B. Further binning of data in Fig. 7. D, based on the diameter of the plaque core marked by Aβ42 staining into 0-10μm, 10-20μm, 20-80μm. C. ELISA results of Aβ42 of 12-month-old hippocampal samples. Statistics calculated using two-tailed unpaired student’s t-tests and ordinary one-way ANOVA using Tukey’s multiple comparison tests with Bartlett’s test correction. D-F. Uncut blots for the proteins involved in UNC5C T835M-mediated apoptotic pathway and loading controls at 6 months and at 12 months with red boxes showing the bands represented in Fig. 7L. Asterisk in NOX1 blot at 6 months indicate non-specific bands and were not used in the analysis and Asterisk in GAPDH blot in panel E shows CDK5 bands what were left after stripping. Data are presented as mean ± SEM. Blue circles - males; pink triangle - females. *n*=5/sex/genotype/timepoint. Only comparisons with significant *p*-value are indicated. * *p*-value ≤ 0.05, ** *p*-value ≤ 0.01, *** *p*-value ≤ 0.001, and **** *p*-value of ≤ 0.0001.

Additional file 7: Supplementary figure S7: Astrocyte morphology is not altered between NLGF and dKI mice. A. Uncut blots for the axonal/synaptic proteins and loading controls with red boxes showing the bands represented in Fig. 8 A. B-B”. Step-wise IMARIS 3D reconstruction showing the morphology of astrocytes in the CA1 region of NLGF and dKI mice at 12 months. Scale bar, 15μm. The original image with astrocytes stained with GFAP, BACE1 and DAPI in NLGFand dKI mice, Image rendered after applying the surface tool to show the astrocytic processes, Further slice rendering under the surface tool to fill the astrocytic processes. C-J. Quantification of astrocytic processes parameters including area, process length, soma area, volume, number of branch terminal points, number of dendritic branches, number of dendritic segmentsand dendritic branch points. *n*=5 females, 5 males/genotype. Statistics calculated using two-tailed unpaired student’s t-tests and ordinary one-way ANOVA using Tukey’s multiple comparison tests with Bartlett’s test correction. Data are presented as mean ± SEM. Only comparisons with significant *p*-value are indicated. * *p*-value ≤ 0.05, ** *p*-value ≤ 0.01, *** *p*-value ≤ 0.001, and **** *p*-value of ≤ 0.0001.

Additional file 8: S8: Excel file with results of TMT-MS Proteomic study on the hippocampal samples of 18-month-old *Unc5c+/+* and *Unc5c*^*KI/KI*^ mice.

## Data Availability

All datasets generated are included in this article and the supplemental information files.
