# Supplementary figures and images for: The UNC5C T835M mutation associated with Alzheimer’s disease leads to neurodegeneration involving oxidative stress and hippocampal atrophy in aged mice
Source: Mol Neurodegener. 2025 Jun 4;20:65. doi: 10.1186/s13024-025-00850-z (PMC12135551; doi:10.1186/s13024-025-00850-z)

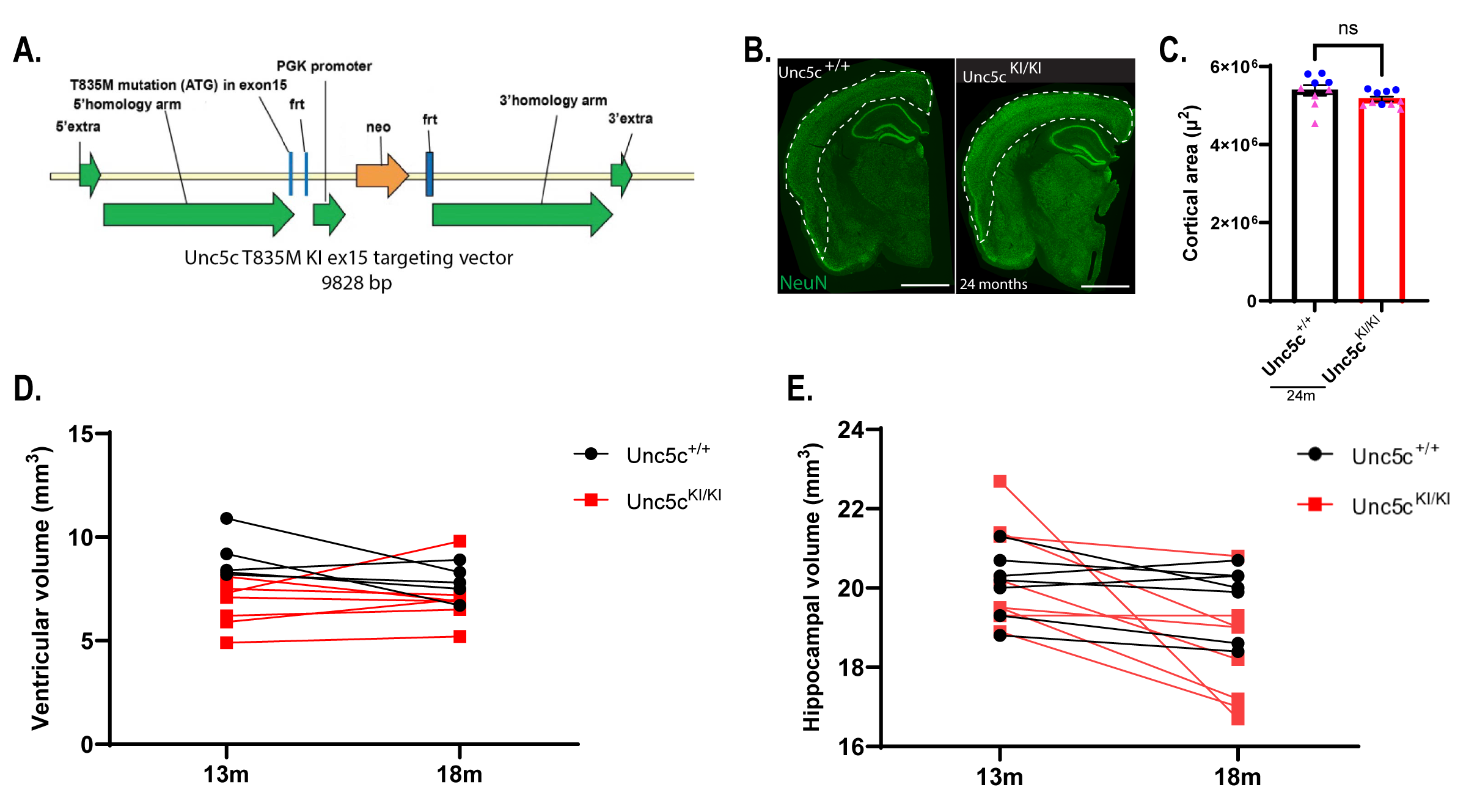

Supplement: Supplementary file 1 — Additional file 1: Supplementary figure S1: Hippocampal neurodegeneration is observed at 18 months in the Unc5cKI/KI mice. A. Schematic representation of UNC5C T835M Exon 15 knock-in targeting vector B. Confocal image highlighting the cortical area with dashed yellow region at 24 months in Unc5c+/+ and Unc5cKI/KImice. Scale bar, 1.16 mm. C. Quantification of cortical area by ImageJ at 24 months. n=7, Unc5c+/+; n=8, Unc5cKI/KI. Blue circles - males; pink triangle - females. D, E. Graph showing the change in volume of ventricles and hippocampus over time for individual animals used in the study. Statistics calculated using two-tailed unpaired student’s t-tests. Data are presented as mean ± SEM. Only comparisons with significant p-value are indicated. * p-value ≤ 0.05, ** p-value ≤ 0.01, *** p-value ≤ 0.001, and **** p-value of ≤ 0.0001. [file 13024_2025_850_MOESM1_ESM.tif]

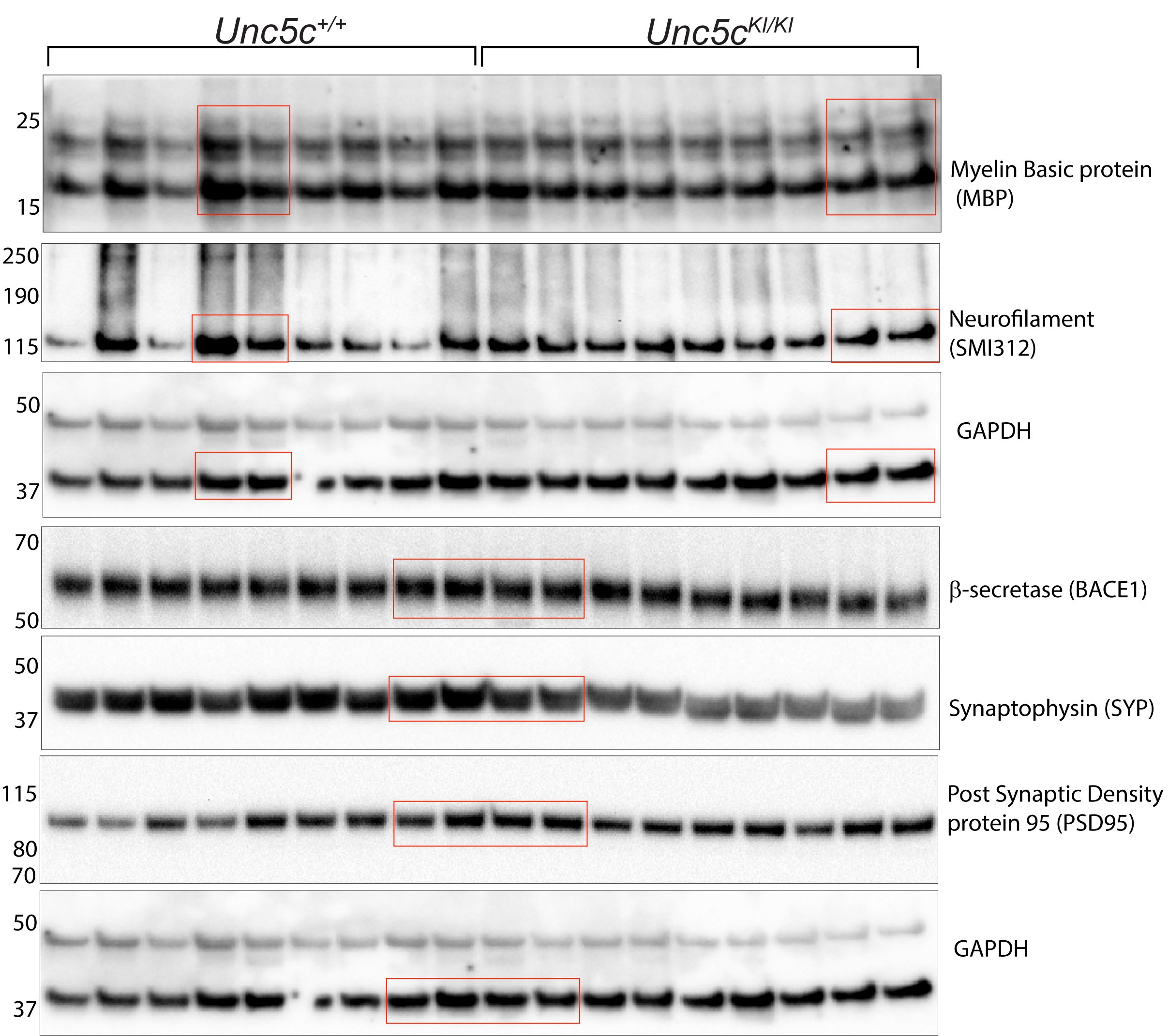

Supplement: Supplementary file 2 — Additional file 2: Supplementary figure S2: Uncut blots for the axonal/pre- and post-synaptic proteins and loading controls with red boxes showing the bands represented in Fig. 2 A. Blots were cut around the protein size to probe the protein of interest. Loading control for each blot used is shown underneath the proteins investigated. [file 13024_2025_850_MOESM2_ESM.tif]

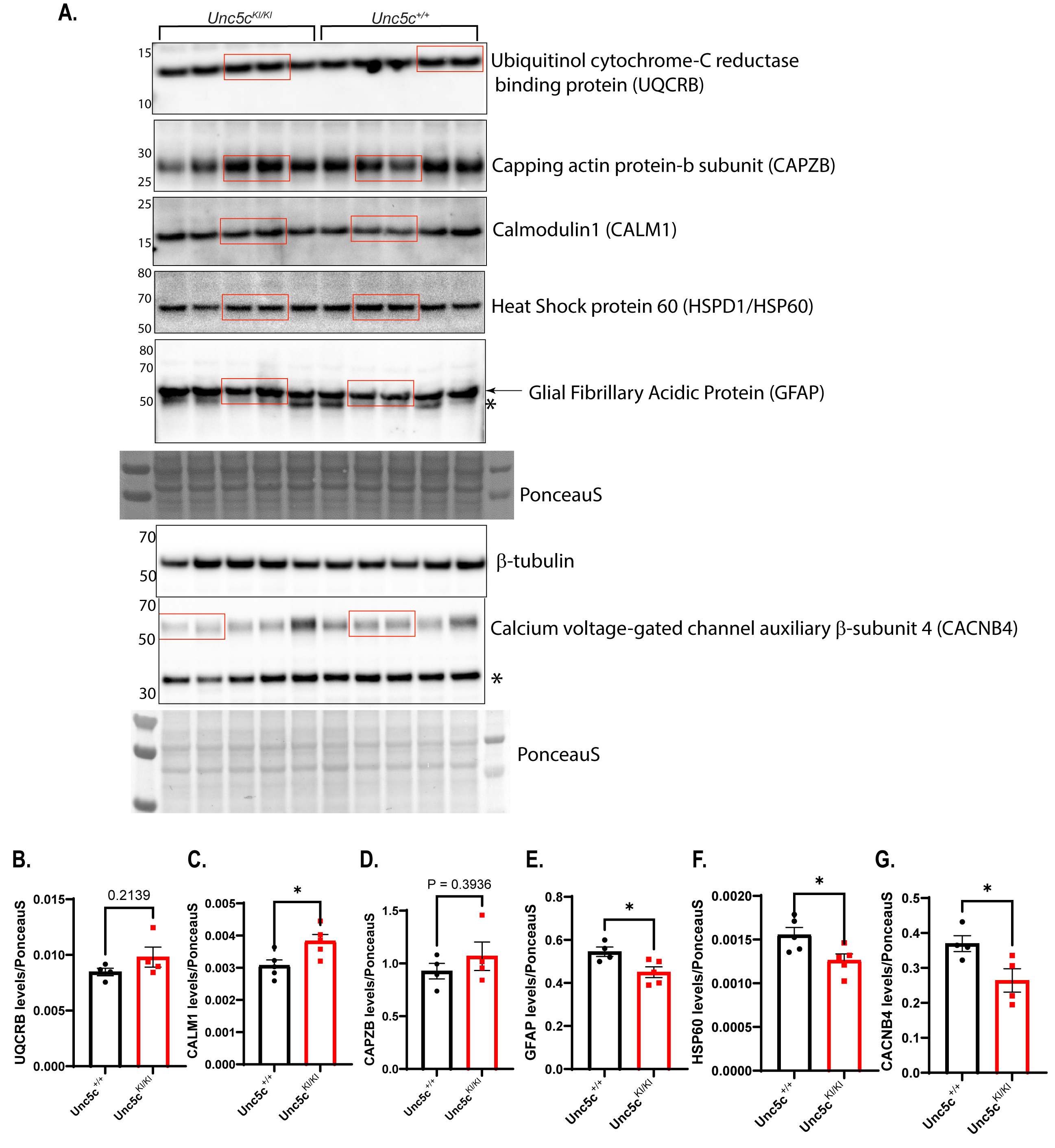

Supplement: Supplementary file 3 — Additional file 3: Supplementary figure S3: A. Uncut blots for the up-regulated and down-regulated proteins loading controls and PonceauS with red boxes showing the bands represented in Fig. 3D, J. Asterisk in GFAP and CACNB4 blots indicate non-specific bands and were not used in the analysis. B-G. Quantification of the immunoblots for UQCRB, CALM1and CAPZB, GFAP, HSPD1/HSP60 and CACNB4 normalized to PonceauS. Statistics calculated using two-tailed unpaired student’s t-tests. Data are presented as mean ± SEM. Only comparisons with significant p-value are indicated. * p-value ≤ 0.05, ** p-value ≤ 0.01, *** p-value ≤ 0.001, and **** p-value of ≤ 0.0001. [file 13024_2025_850_MOESM3_ESM.tif]

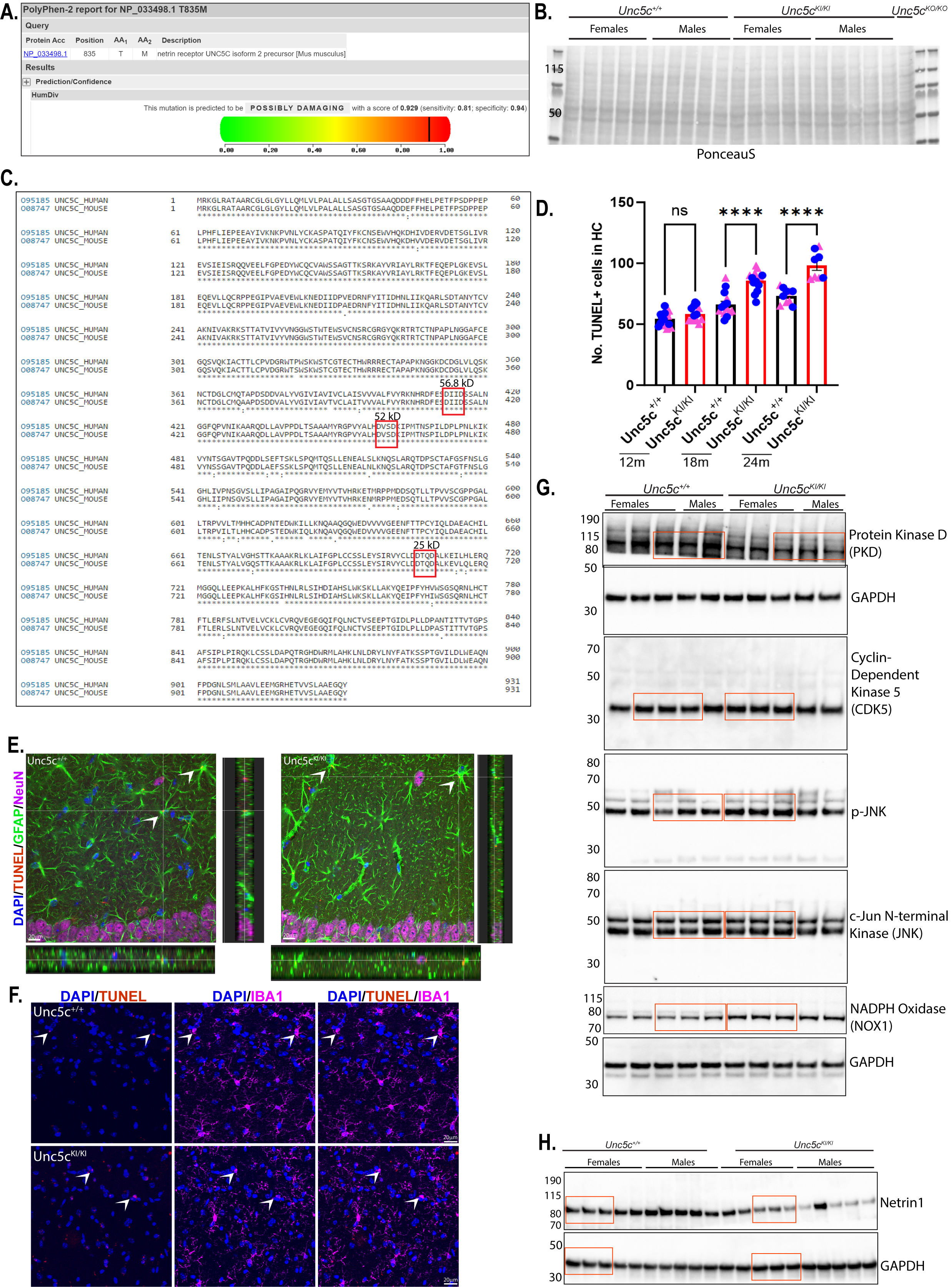

Supplement: Supplementary file 4 — Additional file 4: Supplementary figure S4: Increased neuronal apoptosis in Unc5cKI/KI mice. A. Snapshot of results of Polyphen-2 software showing the effects and score of the T835M mutation on UN5C protein structure and function. B. PonceauS stain showing the loading of Unc5c+/+, Unc5cKI/KI and Unc5cKO/KO hippocampal samples for the UNC5C blot in Fig. 4B. C. Pairwise alignment of the human and mouse UNC5C protein sequences with the predicted Caspase-3 cleavage sites in red boxes and the predicted sizes of the cleaved fragments to be obtained from those cleavage sites are indicated above those boxes. D. Quantification of number of TUNEL+ cells in hippocampal sections of Unc5c+/+ and Unc5cKI/KI mice. Blue circles - males; pink triangle - females. N=5-7 males, n=5-8 females/genotype/age. Statistics calculated using two-tailed unpaired student’s t-tests and ordinary one-way ANOVA using Tukey’s multiple comparison tests with Bartlett’s test correction. E. A single-plane orthogonal view of a confocal image of CA1 region from 18 m Unc5c+/+ and Unc5cKI/KI mice stained for TUNEL-positive cells. Scale bar, 20 μm, F. 20x confocal images of CA1 region from 18 m Unc5c+/+ and Unc5cKI/KI mice stained for TUNEL-positive cells. Scale bar, 20 μm. G, H. Uncut blots for proteins involved in UNC5C T835M-mediated apoptosis, loading control and netrin1, loading control with red boxes showing the bands represented in Fig. 4H. Data are presented as mean ± SEM. Only comparisons with significant p-value are indicated. * p-value ≤ 0.05, ** p-value ≤ 0.01, *** p-value ≤ 0.001, and **** p-value of ≤ 0.0001. [file 13024_2025_850_MOESM4_ESM.tif]

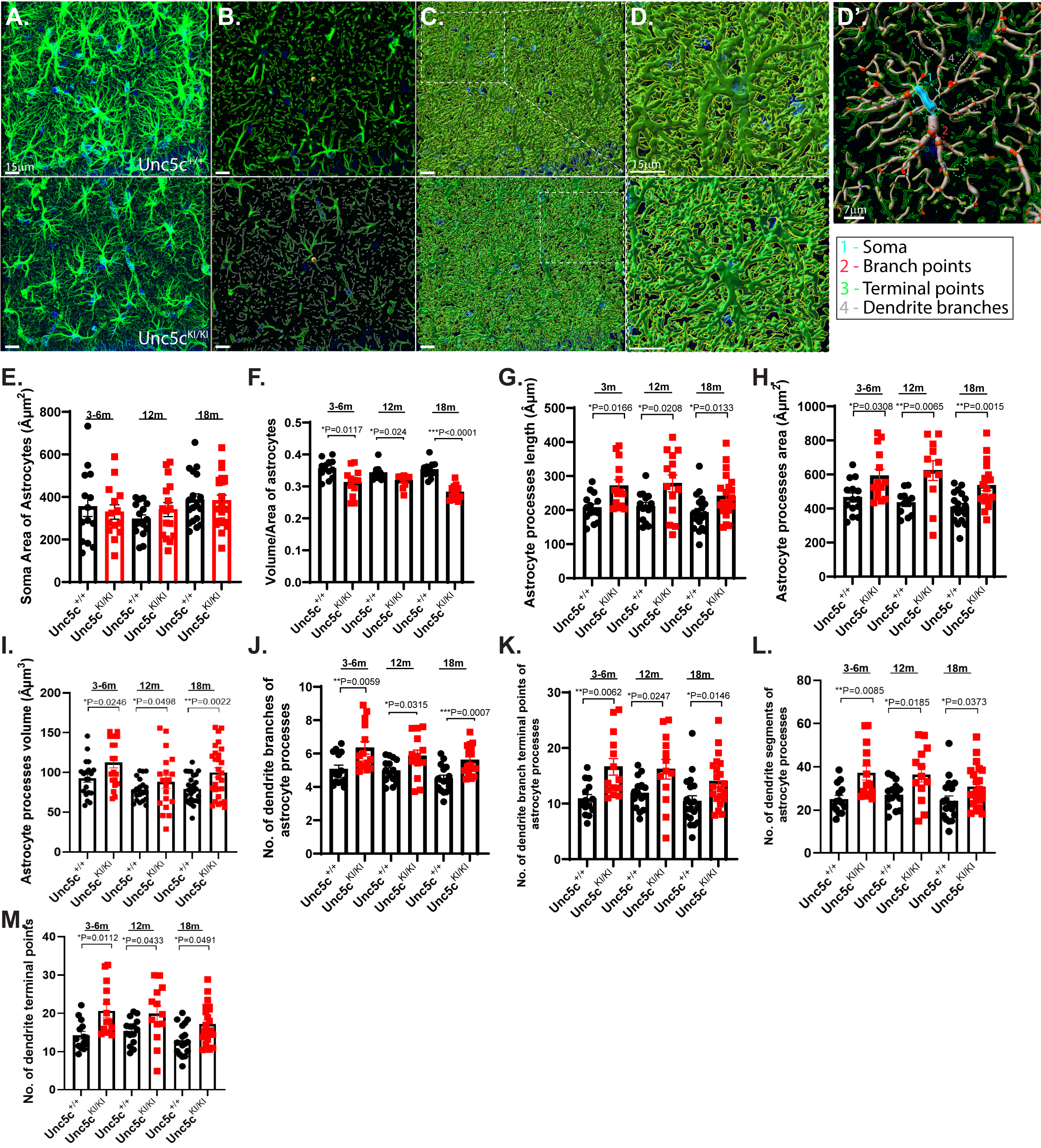

Supplement: Supplementary file 5 — Additional file 5: Supplementary figure S5: Astrocyte morphology is significantly changed in the Unc5cKI/KI mice. A-D’. Step-wise IMARIS 3D reconstruction showing the morphology of astrocytes in the CA1 region of Unc5c+/+ and Unc5cKI/KI mice. Scale bar, 15μm - The original image with astrocytes stained with GFAP and DAPI (A), Image rendered after applying the surface tool to show the astrocytic processes (B), Further slice rendering under the surface tool to fill the astrocytic processes (C), magnified image focusing on a single astrocyte highlighted in the dashed box in B (D), scale bar, 15μm, Filament tool reconstruction of astrocyte circled in D showing different analyzed parameters – soma, branch points, terminal points, dendrite branches, (D') scale bar, 7 μm. E-M. Quantification of astrocytic processes parameters including soma area, volume to area ratio, process length, area, volume, number of dendritic branches, number of branch terminal points, number of dendritic segmentsand dendritic terminal points. n=6-8 females, 5-7 males/genotype/timepoint. Statistics calculated using two-tailed unpaired student’s t-tests and ordinary one-way ANOVA using Tukey’s multiple comparison tests with Bartlett’s test correction. Data are presented as mean ± SEM. Only comparisons with significant p-value are indicated. * p-value ≤ 0.05, ** p-value ≤ 0.01, *** p-value ≤ 0.001, and **** p-value of ≤ 0.0001. [file 13024_2025_850_MOESM5_ESM.tif]

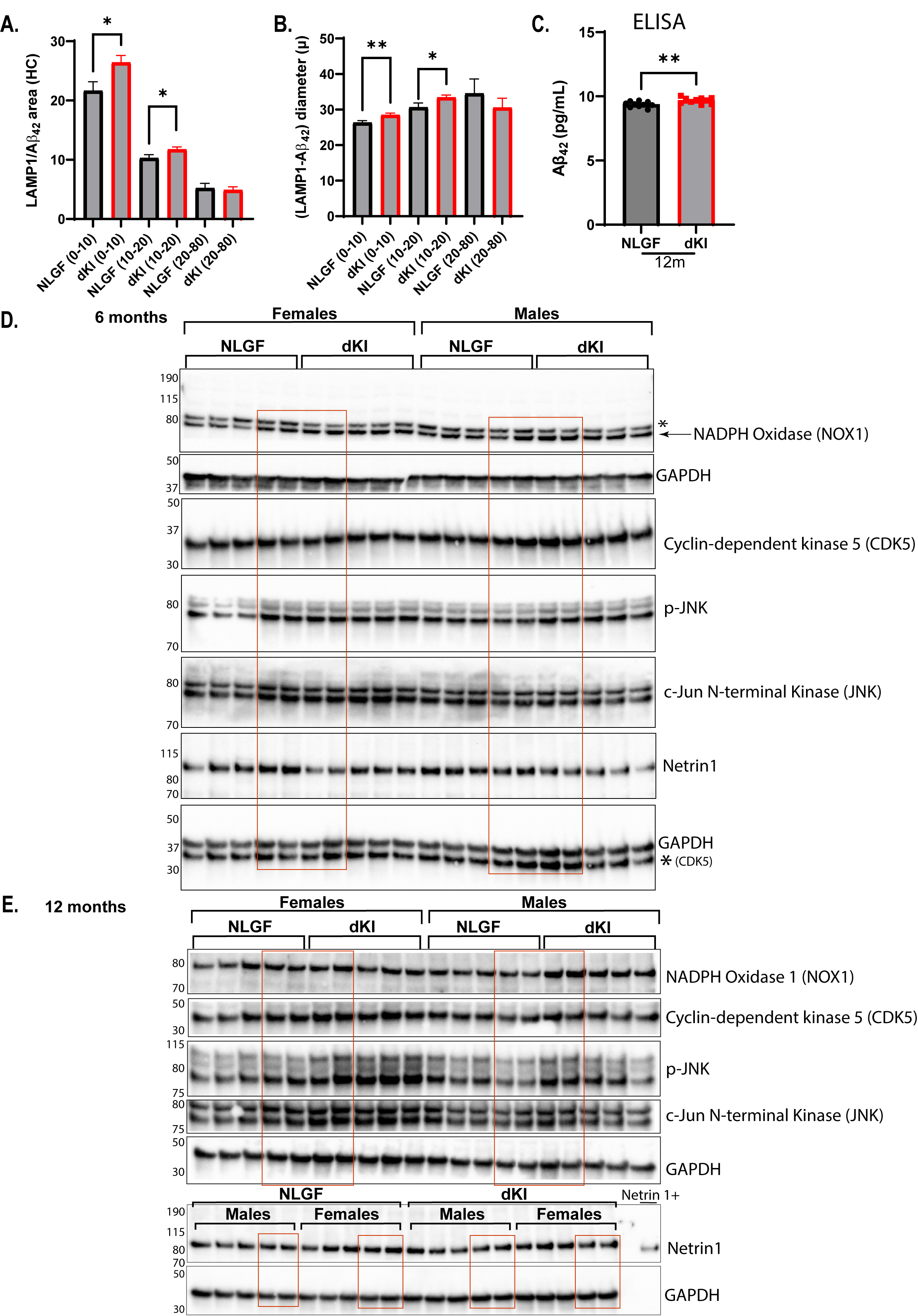

Supplement: Supplementary file 6 — Additional file 6: Supplementary figure S6: Plaque associated dystrophic neurites are inversely proportional to the plaque core. A, B. Further binning of data in Fig. 7. D, based on the diameter of the plaque core marked by Aβ42 staining into 0-10μm, 10-20μm, 20-80μm. C. ELISA results of Aβ42 of 12-month-old hippocampal samples. Statistics calculated using two-tailed unpaired student’s t-tests and ordinary one-way ANOVA using Tukey’s multiple comparison tests with Bartlett’s test correction. D-F. Uncut blots for the proteins involved in UNC5C T835M-mediated apoptotic pathway and loading controls at 6 months and at 12 months with red boxes showing the bands represented in Fig. 7L. Asterisk in NOX1 blot at 6 months indicate non-specific bands and were not used in the analysis and Asterisk in GAPDH blot in panel E shows CDK5 bands what were left after stripping. Data are presented as mean ± SEM. Blue circles - males; pink triangle - females. n=5/sex/genotype/timepoint. Only comparisons with significant p-value are indicated. * p-value ≤ 0.05, ** p-value ≤ 0.01, *** p-value ≤ 0.001, and **** p-value of ≤ 0.0001. [file 13024_2025_850_MOESM6_ESM.tif]

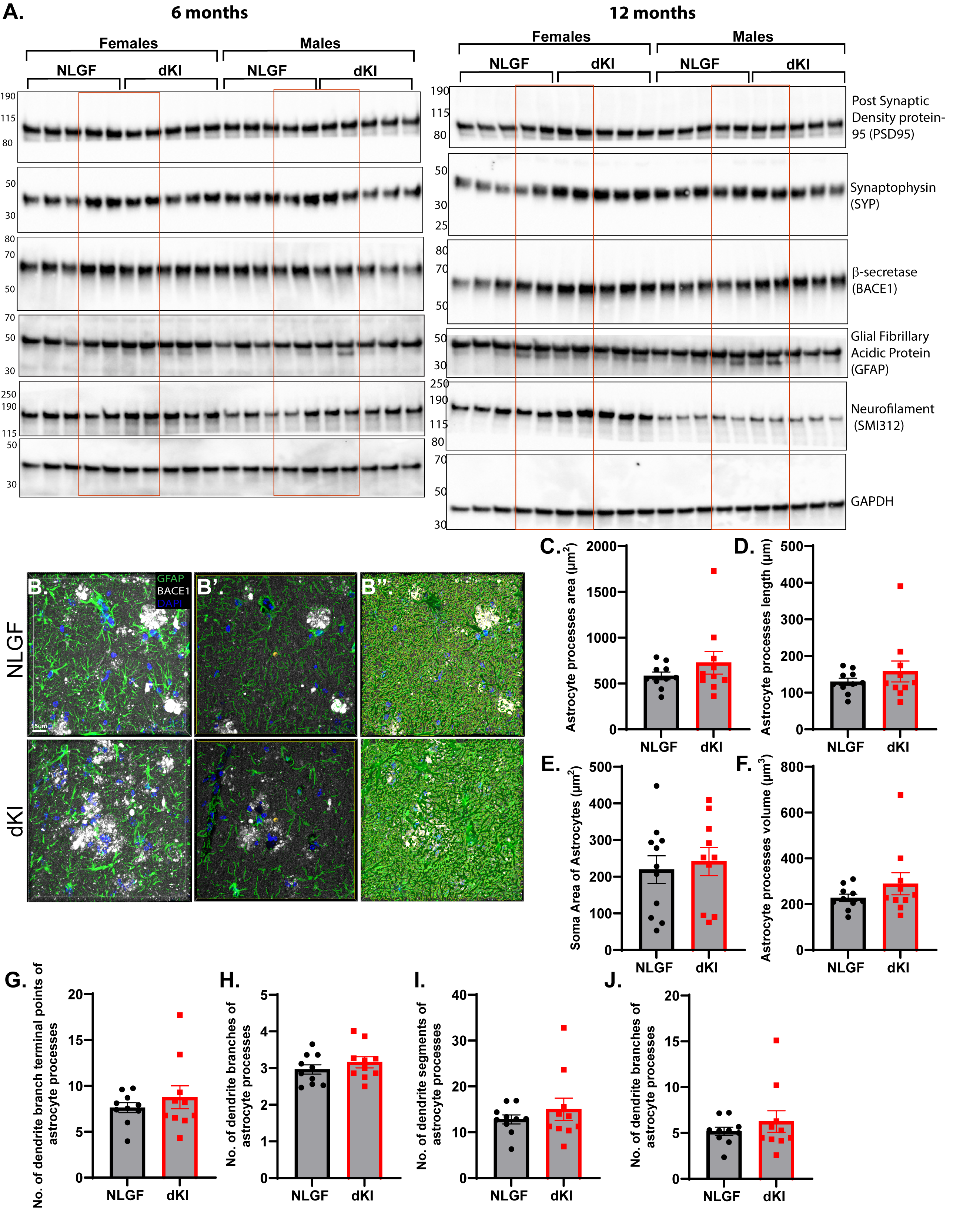

Supplement: Supplementary file 7 — Additional file 7: Supplementary figure S7: Astrocyte morphology is not altered between NLGF and dKI mice. A. Uncut blots for the axonal/synaptic proteins and loading controls with red boxes showing the bands represented in Fig. 8 A. B-B”. Step-wise IMARIS 3D reconstruction showing the morphology of astrocytes in the CA1 region of NLGF and dKI mice at 12 months. Scale bar, 15μm. The original image with astrocytes stained with GFAP, BACE1 and DAPI in NLGFand dKI mice, Image rendered after applying the surface tool to show the astrocytic processes, Further slice rendering under the surface tool to fill the astrocytic processes. C-J. Quantification of astrocytic processes parameters including area, process length, soma area, volume, number of branch terminal points, number of dendritic branches, number of dendritic segmentsand dendritic branch points. n=5 females, 5 males/genotype. Statistics calculated using two-tailed unpaired student’s t-tests and ordinary one-way ANOVA using Tukey’s multiple comparison tests with Bartlett’s test correction. Data are presented as mean ± SEM. Only comparisons with significant p-value are indicated. * p-value ≤ 0.05, ** p-value ≤ 0.01, *** p-value ≤ 0.001, and **** p-value of ≤ 0.0001. [file 13024_2025_850_MOESM7_ESM.tif]
